# A pathological joint–liver axis mediated by matrikine-activated CD4^+^ T cells

**DOI:** 10.1038/s41392-024-01819-y

**Published:** 2024-05-08

**Authors:** Junzhi Yi, Hui Zhang, Fangyuan Bao, Zhichu Chen, Yuliang Zhong, Tianning Ye, Xuri Chen, Jingyi Qian, Mengya Tian, Min Zhu, Zhi Peng, Zongyou Pan, Jianyou Li, Zihao Hu, Weiliang Shen, Jiaqi Xu, Xianzhu Zhang, Youzhi Cai, Mengjie Wu, Hua Liu, Jing Zhou, Hongwei Ouyang

**Affiliations:** 1 Department of Sports Medicine of the Second Affiliated Hospital, and Liangzhu Laboratory, Zhejiang University School of Medicine, Hangzhou, China; 2 Dr. Li Dak Sum & Yip Yio Chin Center for Stem Cells and Regenerative Medicine, Zhejiang University School of Medicine, Hangzhou, China; 3 Stomatology Hospital, School of Stomatology, Zhejiang University School of Medicine, Zhejiang Provincial Clinical Research Center for Oral Diseases, Hangzhou, China; 4 The MOE Key Laboratory of Biosystems Homeostasis & Protection, The Key Laboratory of Cancer Molecular Cell Biology of Zhejiang Province, Life Sciences Institute, Zhejiang University, Hangzhou, China; 5 Department of Orthopedics, Huzhou Central Hospital, Affiliated Huzhou Hospital, Zhejiang University School of Medicine, Huzhou, China; 6 China Orthopedic Regenerative Medicine Group (CORMed), Hangzhou, China; 7 Department of Orthopedics, The First Affiliated Hospital, Zhejiang University School of Medicine, Hangzhou, China

**Keywords:** Immunological disorders, Preclinical research

## Abstract

The knee joint has long been considered a closed system. The pathological effects of joint diseases on distant organs have not been investigated. Herein, our clinical data showed that post-traumatic joint damage, combined with joint bleeding (hemarthrosis), exhibits a worse liver function compared with healthy control. With mouse model, hemarthrosis induces both cartilage degeneration and remote liver damage. Next, we found that hemarthrosis induces the upregulation in ratio and differentiation towards Th17 cells of CD4^+^ T cells in peripheral blood and spleen. Deletion of CD4^+^ T cells reverses hemarthrosis-induced liver damage. Degeneration of cartilage matrix induced by hemarthrosis upregulates serological type II collagen (COL II), which activates CD4^+^ T cells. Systemic application of a COL II antibody blocks the activation. Furthermore, bulk RNAseq and single-cell qPCR analysis revealed that the cartilage Akt pathway is inhibited by blood treatment. Intra-articular application of Akt activator blocks the cartilage degeneration and thus protects against the liver impairment in mouse and pig models. Taken together, our study revealed a pathological joint–liver axis mediated by matrikine-activated CD4^+^ T cells, which refreshes the organ-crosstalk axis and provides a new treatment target for hemarthrosis-related disease.

**Figure:**
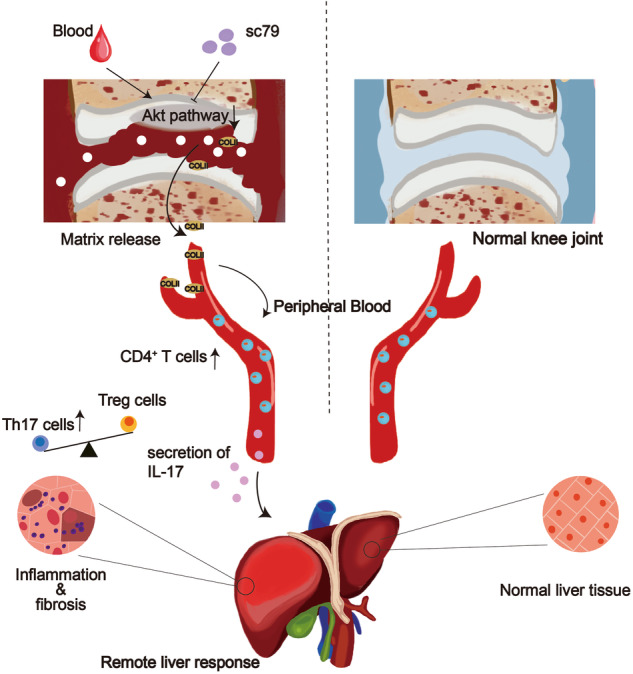
Intra-articular bleeding induces cartilage degradation through down-reulation of cartilage Akt pathway. During this process, the soluble COL II released from the damaged cartilage can activate peripheral CD4^+^ T cells, differention into Th17 cells and secretion of IL-17, which consequently induces liver impairment. Intra-articular application of sc79 (inhibitor of Akt pathway) can prevent the cartilage damage as well as its peripheral influences.

Intra-articular bleeding induces cartilage degradation through down-reulation of cartilage Akt pathway. During this process, the soluble COL II released from the damaged cartilage can activate peripheral CD4^+^ T cells, differention into Th17 cells and secretion of IL-17, which consequently induces liver impairment. Intra-articular application of sc79 (inhibitor of Akt pathway) can prevent the cartilage damage as well as its peripheral influences.

## Introduction

Post-traumatic osteoarthritis (OA) develops after post-traumatic joint damage and accounts for 12% of all cases of symptomatic osteoarthritis.^1^ Intra-articular (IA) joint bleeding (hemarthrosis) plays an important role in the pathophysiology of post-traumatic joint damage.^2^ In addition, hemarthrosis leads to severe joint damage and instability in patients with hemophilia and total knee arthroplasty.^3,4^ The articular cartilage shows poor capability for endogenous repair, while IA blood can lead to cartilage degradation both directly and through the joint microenvironment. After hemarthrosis, iron is deposited in the superficial and subsynovial layers of the synovium, leading to the hyperplasia and hypertrophy, fibrosis, neovascularization of synovium.^2^ The blood-treated synovium increases the secretion of pro-inflammatory cytokines^5^ and matrix-degrading proteinases to damage joint microenvironment and cartilage.^6^ In addition, cartilage-destructive factors can be induced in the blood-treated cartilage itself^7^ and erythrocyte-derived iron can lead to direct chondrocyte apoptosis through the production of hydroxyl radicals.^8,9^ Though these micro-environmental factors have been confirmed, the alterations of signal transduction in cartilage tissue in a hemarthrosis environment are poorly understood.

Recently, the crosstalks between various distant organs and tissue in the body have been widely studied. Our previous study found that biomaterials implanted into the abdominal wall cause liver fatty deposition;^10^ Inflammatory disorders of the skin contribute to inflammatory bowel diseases.^11^ As the knee joint has the structure of menisci and synovium sealing the joint cavity, and the articular cartilage has its avascular and aneural nature, the knee joint is regarded as a closed system. The crosstalks between cartilage damage and responses in distant organ are still not clear. Nevertheless, previous studies have found some serological biomarkers, including soluble extracellular matrix (ECM) and inflammatory and metabolic mediators, whose levels are significantly increased during joint damage in hemophilic arthropathy and OA.^12,13^ Notably, the solid organ can be influenced by soluble ECM (also called matrikines) in the peripheral blood. For example, skin-derived fragmented hyaluronic acid (HA) can cause distant inflammatory bowel diseases,^11^ the circulating elastin-derived peptides (EDPs) can cause non-alcoholic fatty liver disease (NAFLD).^14^ In addition, soluble ECM can induce immune responses and immune cell activation. For example, the release of collagen fragments and elastin fragments has been reported to lead to neutrophil-mediated pulmonary inflammation;^15,16^ the release of soluble heparan sulfate (HS) contributes to the secretion of pro-inflammatory factors by peripheral mononuclear cells and splenocytes.^17^ As excessive inflammation can lead to tissue damage, it is possible that the soluble ECM in peripheral blood may induce a solid organ response by activating the immune response.

Regarding the specific soluble ECM, immune response and distant organ damage, COL II is the major structural protein of cartilage, meanwhile studies have found the upregulation of soluble COL II (sCOL II) in serum during hemophilic arthropathy^18^ and OA.^13^ However, the biological effects of joint damage-induced upregulation of serological COL II remain unknown. T helper (CD4^+^ T) cells can be activated by several self-produced components and are involved in autoimmune diseases.^19,20^ Dysfunctions of CD4^+^ T cells guided by antigen-processing cells and/or an inflammatory microenvironment contribute to liver inflammation, fibrosis and fatty liver disease.^21^ Therefore, it is possible that CD4^+^ T cells are activated by self-produced sCOL II during arthropathy and then cause liver disease.

Here, we collected data from patients with post-traumatic joint damage, primary OA and normal joint, revealing a crosstalk between the liver and joint suffered from trauma. Using a mouse model of hemarthrosis, we explained the pathological joint–liver axis. Hemarthrosis induces cartilage degeneration and COL II release through inhibition of the cartilage Akt pathway. The cartilage-released serological COL II activates CD4^+^ T cells, resulting in liver damage. The findings refresh organ-crosstalk phenomenon in the body and provide a new target for the treatment of hemarthrosis-related diseases.

## Results

### Patients with post-traumatic joint damage (PTJD) have a higher chance of abnormal liver function

**Fig. 1:**
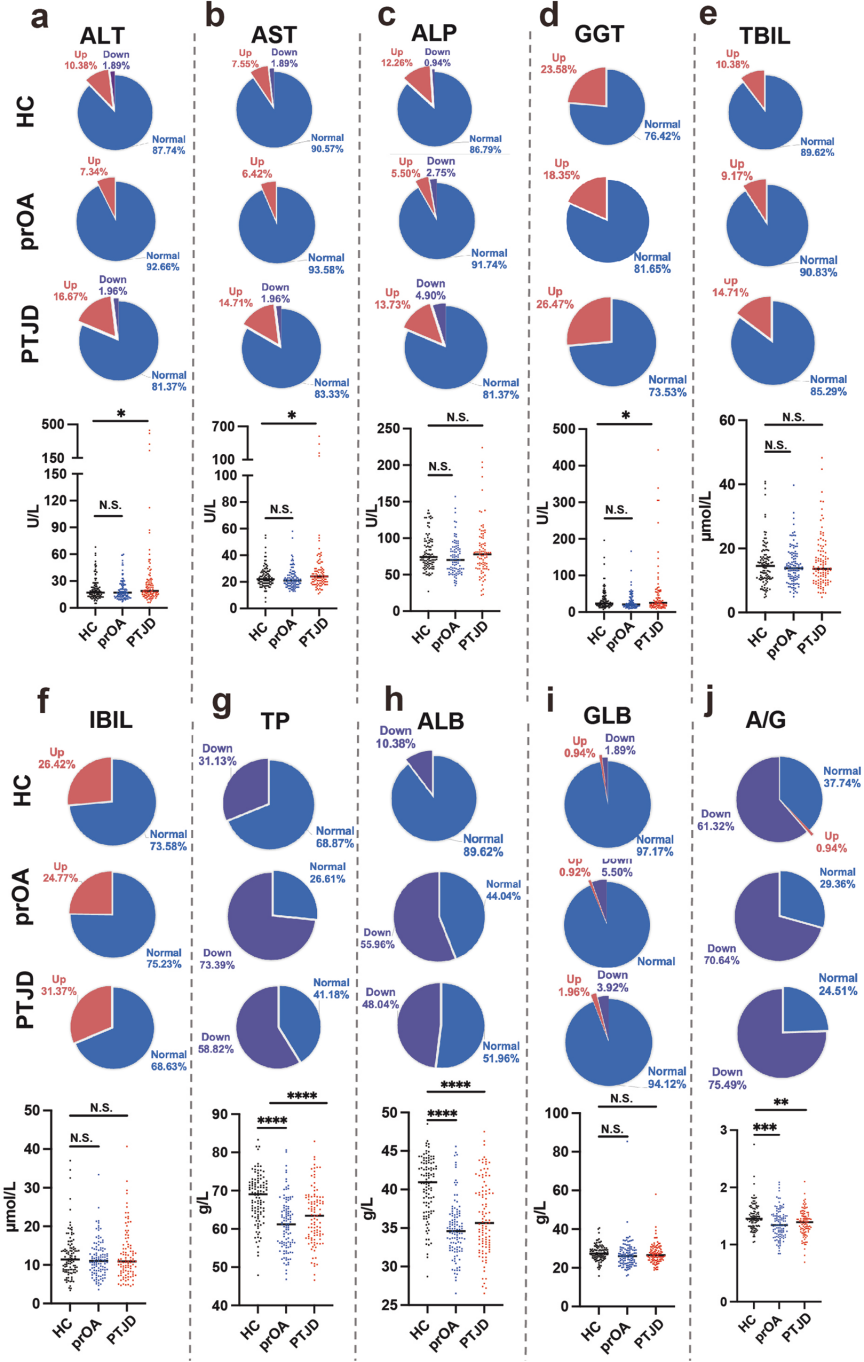
Post-traumatic joint damage (PTJD) patients show worse liver function. The serological markers of liver function, including **a** ALT (*n* = 102–109), **b** AST (*n* = 101–108), **c** ALP (*n* = 87–99), **d** GGT (*n* = 84–98), **e** TBIL (*n* = 101–109), **f** IBIL (*n* = 101–109), **g** TP (*n* = 102–109), **h** ALB (*n* = 102–109), **i** GLB (*n* = 102–109), and **j** A/G (*n* = 102–109) were showed by both pie charts for the ratios of normal values and bar plots for the absolute values. *P < 0.05, **P < 0.01, ***P < 0.001, ****P < 0.0001

To investigate the relationship between joint damage and systemic response, we collected serum biochemical data from PTJD, primary OA (prOA) and healthy controls (HC) for evaluation of liver function index (Supplementary Table 1 for the baseline characteristics), including alanine transaminase (ALT), aspartate aminotransferase (AST), alkaline phosphatase (ALP), γ-glutamyl transpeptidase (GGT), total bilirubin (TBIL), indirect bilirubin (IBIL), total protein (TP), albumin (ALB), globulin (GLB) and A/G (albumin/globulin). For ALT, AST, and GGT, the ratios of normal values were the lowest in PTJD among the three groups (pie charts), and PTJD patients showed significantly higher absolute levels (bar plots) compared with the HC group, while prOA did not show a consistent trend (Fig. 1a, b, d). For ALP, TBIL, and IBIL, although PTJD did not show significant changes in absolute levels (bar plots), all the ratios of normal value were also the lowest in PTJD among the 3 groups (pie charts), while the ratios of those of prOA were even higher than those of HC group (Fig. 1c, e, f). For TP, ALB, and A/G, both PTJD and prOA groups not only showed lower ratios of normal values (pie charts) but also showed significantly lower absolute values (bar plots) compared with the HC group (Fig. 1g, h, j). All these three groups showed similar GLB level and the ratio of normal value (Fig. 1i). Collectively, these data suggested that PTJD leads to a higher probability of worse liver function while prOA induces different indices with different trends.

### A mouse model of joint bleeding (hemarthrosis) reveals both local cartilage and remote liver damage

**Fig. 2:**
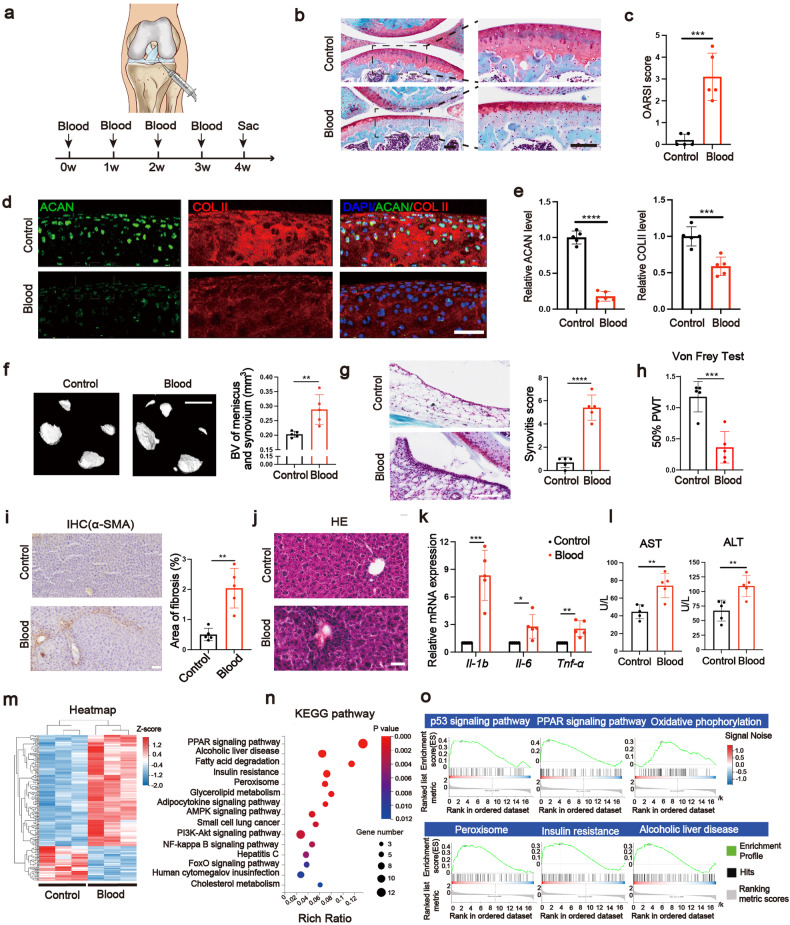
A hemarthrosis mice model reveals both local cartilage and remote liver damage. **a** The experimental procedure for blood treatment in vivo. Mice were intra-articular injected with blood every week for four times. **b** SO staining of knee joints (scale bar, 100 µm). **c** Quantification of the SO staining with OARSI score (*n* = 5). **d**, **e** Immunofluorescence analysis of ACAN (green) and COL II (red) of articular cartilage treated with blood (scale bar, 65 µm) and the quantitation (*n* = 5). **f** After the IA blood treatment, calcification of meniscus and synovium were assayed by corresponding bone volume (BV) (scale bar, 800 µm) (*n* = 5). **g** SO staining of the collected synovium and corresponding Synovitis score (scale bar, 120 µm) (*n* = 5). **h** Hyperalgesia was assayed by Von Frey Test (*n* = 5). **i** IHC analysis on α-SMA of the collected liver (scale bar, 50 µm) and the fibrosis areas were quantified (*n* = 5). **j** HE staining of the collected liver (scale bar, 40 µm) (*n* = 5). **k** The liver mRNA expression of *Il-1b*, *Il-6*, and *Tnf-α* measured by qPCR (*n* = 5). **l** Serum biochemical analysis of AST and ALT (*n* = 5). **m** Heatmap of liver tissue from RNA-seq data (*n* = 3). **n** Kyoto Encyclopedia of Genes Genomes (KEGG) pathway analysis from upregulated genes of blood-treated liver tissue (*n* = 3). **o** Gene Set Enrichment Analysis (GSEA) of upregulated pathways in Blood group (*n* = 3). *P < 0.05, **P < 0.01, ***P < 0.001, ****P < 0.0001

Since one of the differences between patients with post-traumatic joint damage and primary OA is that the former also suffer from joint bleeding, we then investigated the effects of blood on the remote organ response. A mouse model of hemarthrosis (Fig. 2a) was established as described in “Methods”. After the injection, Blood group showed significant cartilage degeneration and synovial inflammation and hyperplasia in comparison with the Control group (Fig. 2b, c, g). The protein levels of ACAN and COL II in cartilage areas were significantly suppressed in Blood group (Fig. 2d, e). We also revealed a significant increase of hyperalgesia, calcification of meniscus and synovium, and subchondral bone sclerosis but no significant increase of osteophyte formation in Blood group (Fig. 2f, h and Supplementary Fig. 1a, b). During in vitro experiments (Supplementary Fig. 2a), the chondrocyte viability and the level of apoptosis-resistant *Bcl-2* were significantly decreased as being treated with 5% and 10% blood (Supplementary Fig 2g, h). We also observed the downregulation of *Acan* and *Col II* as being treated with blood at both protein and mRNA levels (Supplementary Fig. 2b, d, e). Apoptosis-promoting cleaved caspase-3 (CASP3, protein level) and *Bax* (mRNA level) as well as matrix-degradation-related *Mmp3* and *Mmp13* (mRNA level) were significantly increased in Blood group (Supplementary Fig 2c, f, h, i). Then, we detected liver function at different time points (Supplementary Figs. 3 and 2i–o). We found significant liver impairment in the Blood group at week 4 (after 4 times of IA blood injection) as evidenced by significantly increased serum biochemical indicators (ALT and AST), expression of inflammatory marker genes (*Il-1b*, *Il-6*, and *Tnf-*α) as well as fibrosis (α-SMA) areas (Fig. 2i–l). At week 16, the liver impairment was still not recovered and lipid deposition was found in liver tissue (Supplementary Fig. 3a–c). Before week 4, only at week 3 could slightly liver impairment be detected (Supplementary Fig. 3a–c). Therefore, week 4 was chosen as the time point for further experiments. We further clarified the global insights of liver damage by performing RNA-seq. The heatmap showed the 165 upregulated genes and 51 downregulated genes in the Blood group in comparison with the Control group (Fig. 2m). The Kyoto Encyclopedia of Genes and Genomes (KEGG) pathway analysis demonstrated that several steatosis and inflammation-related terms such as PPAR signaling pathway, alcoholic liver disease, insulin resistance, peroxisome, adipokine signaling pathway and NF-κB signaling pathway were among the top 15 upregulated signaling pathways in Blood group (Fig. 2n). The Gene Set Enrichment Analysis (GSEA) results showed similar outcomes, involving upregulated steatosis and apoptosis-related terms in Blood group (Fig. 2o). Overall, we found that IA blood not only damages joint but also induces liver impairment in the mouse model.

### CD4^+^ T cells mediate hemarthrosis-induced liver impairment

**Fig. 3:**
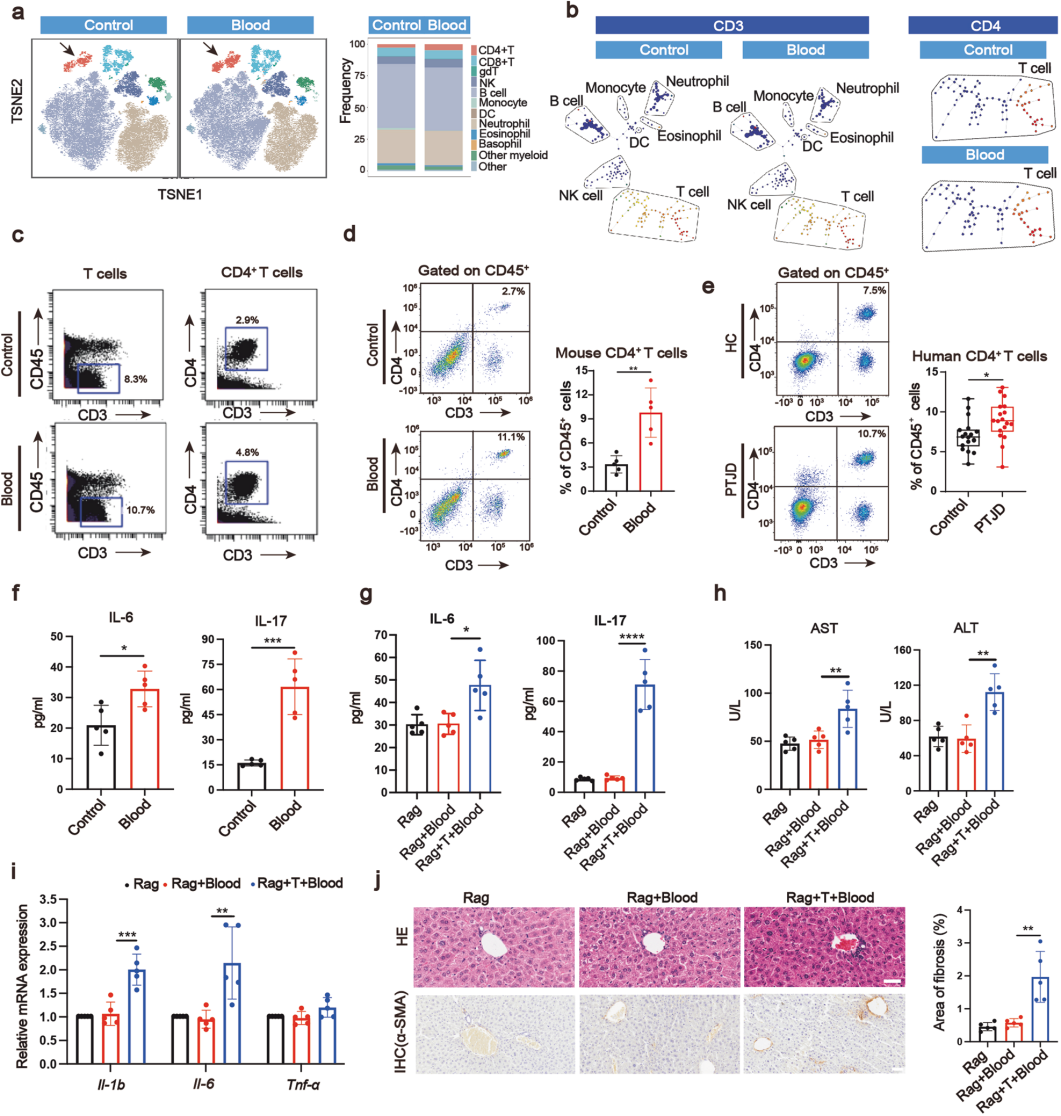
CD4^+^ T cells mediate the cartilage-liver crosstalk. **a** TSNE analysis of CD45^+^ PBMCs from CyTOF data and its corresponding quantification. The arrows point out CD4^+^ T cells. **b**, **c** SPADE analysis of total cells highlighting relative location and content of CD3^+^ cells in them (left panel) and T cells highlighting CD4^+^ cells in them (right panel) and **c** corresponding quantification. **d**, **e** Flow cytometric measurement of the percentages of CD4^+^ T cells in mice and human (*n* = 5 for mice, *n* = 18 for human). **f** ELISA analysis of serum IL-6 and IL-17 protein levels in wild type mice of Blood and Control groups (*n* = 5). **g** ELISA analysis of serum IL-6 and IL-17 levels in Rag1^−/−^ mice (*n* = 5). **h** Serum biochemical analysis of AST and ALT (*n* = 5). **i** The liver mRNA expression of *Il-1b*, *Il-6*, and *Tnf-α* measured by qPCR (*n* = 5). **j** HE staining (scale bar, 40 µm), IHC analysis (scale bar, 50 µm) of α-SMA protein and corresponding quantification of fibrosis areas in collected liver (*n* = 5). In CyTOF analysis, 136389 CD45^+^ cells from four donor mice in Control group and 147281 CD45^+^ cells from four donor mice in Blood group were measured. *P < 0.05, **P < 0.01, ***P < 0.001

Since the joint and the liver are not in direct contact, we next hypothesized that peripheral blood contributes to the crosstalk. Single-cell mass cytometry (CyTOF) was applied to the CD45^+^ peripheral blood mononuclear cells (PBMCs). Using t-Distributed Stochastic Neighbor Embedding (TSNE) analysis, thirty-two cell clusters were identified (Supplementary Fig. 4a), located in ten types of major immune cells (Fig. 3a), with some representative markers listed in Supplementary Fig 4b. Among them, utilizing TSNE and SPADE (spanning tree progression analysis of density-normalized events) analysis, we found that the ratio of CD4^+^ T cells was higher and the ratio of monocytes was lower in Blood group compared with Control group (Fig. 3a–c and Supplementary Fig. 4c). The ratios of other cell types were not obviously changed (Fig. 3a–c and Supplementary Fig. 4c). Flow cytometric measurement of CD4^+^ T cells in mouse peripheral blood, mouse spleen and human peripheral blood also showed the significant increase after hemarthrosis (Fig. 3d, e and Supplementary Fig. 5a). We further detected the subclusters of CD4^+^ T cells in peripheral blood and spleen of mice, revealing an increased ratio of Th17 cells and a decreased ratio of Treg cells in Blood group (Supplementary Fig. 5b). The serum levels of IL-17 and IL-6 were significantly increased in Blood group (Fig. 3f). Application of IL-17 antibody could reverse the upregulation of serological AST and ALT level in vivo compared with Blood group (Supplementary Fig. 6a). We collected the serum from Control or Blood group, to which IL-17 antibody was further added, to add into the media of hepatocytes (AML12 cells). IL-17 antibody reserved the upregulation of the level of inflammation and fibrosis-related gene in hepatocytes induced by serum from Blood group (Supplementary Fig. 6b). To further investigate the function of CD4^+^ T cells in the joint–liver axis, we utilized Rag1^−/−^ mice (Rag), which lack T and B cells, and the Rag1^-/-^ mice adoptively transferred with CD4^+^ T cells (Supplementary Fig. 7a). The sports ability and OARSI score (cartilage damage) of mice with both IA blood treatment and the cell transfer (Rag+T+Blood) were similar to those of mice treated with IA blood alone (Rag+Blood) (Supplementary Fig. 7b–d). Compared with Rag+Blood group, the Rag+T+Blood group showed significantly increased serological levels of IL-6 and IL-17 along with ALT and AST (Fig. 3g, h). The infiltration of immune cells around hepatic vessels and a significant increase in mRNA expression of inflammatory markers (*Il-1b* and *Il-6*) in liver tissues were observed in Rag+Blood+T group compared with Rag+Blood group (Fig. 3i, j). The Rag+Blood+T group also showed a significant increase in fibrosis areas (Fig. 3j). Adoptive transfer of CD4^+^ T cells, when in a normal state (without IA blood treatment), do not harm to the liver (data not show). Taken together, we revealed that CD4^+^ T cells are activated during hemarthrosis, while systemic deletion of CD4^+^ T cells blocks hemarthrosis-induced liver abnormality.

### sCOL II from damaged cartilage induces the activation of CD4^+^ T cells

**Fig. 4:**
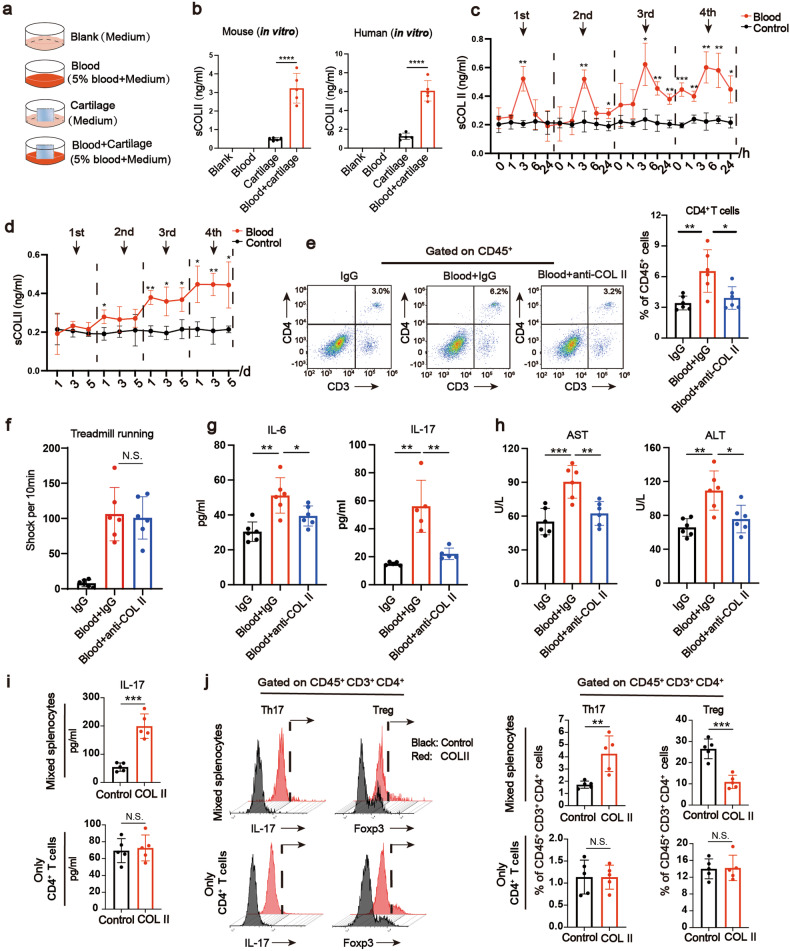
Cartilage-derived sCOL II mediates the systemic activation of CD4 + T cells. **a** The group information for sCOL II detection in vitro. **b** sCOL II level in the supernatant of treated human and mice cartilage explants by ELISA assay (*n* = 5). **c** ELISA analysis of serological COL II level after 1, 3, 6, 24 hours of the 4-time blood treatment (*n* = 3). **d** ELISA analysis of serological COL II level after 1, 3, 5 days of the 4-time blood treatment (*n* = 3). **e** Flow cytometric measurement of the percentages of CD4^+^ T cells in peripheral blood (*n* = 6). **f** The shock times in a 10-minute treadmill running assay (*n* = 6). **g** ELISA analysis of serological IL-6 and IL-17 protein level (*n* = 6). **h** Serum biochemical analysis of AST and ALT (*n* = 6). **i** ELISA analysis of IL-17 protein level of cultural supernatant in “Mixed” group (mixed splenocytes) or “Only” group (only CD4^+^ T cells) (*n* = 5). **j** FACS analysis for Th17 and Treg cells in vitro and the corresponding quantitation (*n* = 5). *P < 0.05, **P < 0.01, ***P < 0.001

Having confirmed the role of CD4^+^ T cells, we then explored how CD4^+^ T cells were activated and the effects of COL II in the soluble state. For in vitro experiments (Fig. 4a), sCOL II level in the culture supernatant of both blood-treated human and mouse explants were significantly increased compared with corresponding cartilage alone (Fig. 4b). For in vivo experiments, the serological COL II analysis was performed at 1, 3, 6 hours and 1, 3, 5 d after each blood treatment. At 3 h after each blood treatment, the level of serological COL II briefly bursted and then decreased (Fig. 4c). Similar with the data that liver started to be slightly damaged (Supplementary Fig. 3), after the third IA blood treatment, a significant decrease of sports ability, stable and significant increase of serological COL II level were observed compared to Control group (Fig. 4c, d). In order to figure out the effects of serological COL II on CD4^+^ T cells, we first utilized anti-COL II antibodies (anti-COL II) and utilized IgG as a control. The sports ability of mice with IA blood and anti-COL II treatment (Blood+anti-COL II) were similar with mice with IA blood and IgG treatment (Blood+IgG) (Fig. 4f). Peripheral CD4^+^ T cells were significantly less activated after the sCOL II was blocked (Fig. 4e). Anti-COL II also significantly reversed the increased serological levels of IL-6 and IL-17 as well as ALT and AST (Fig. 4g, h). To determine whether sCOL II could directly activate CD4^+^ T cells, we extracted mixed splenocytes or only CD4^+^ T cells sorted from splenocytes from the hemarthrosis model. We found that, in the “Mixed” group but not in the “Only” group, the sCOL II could increase the ratio of Th17 cells, decrease the ratio of Treg cells meanwhile upregulate IL-17 content in the culture supernatant (Fig. 4i, j). The sCOL II also upregulated the key stimulator (p-STAT3) of Th17 cells^22^ in CD4^+^ T cells of “Mixed” group (Supplementary Fig. 6c). To further confirm the roles of sCOL II in immune-induced liver impairment, conditioned media from co-cultured sCOL II and mixed PBMCs were utilized to treat hepatocytes (Supplementary Fig. 6d), which resulted in significantly increased expression of inflammation and fibrosis-related genes compared with those of conditioned media from each one (Supplementary Fig. 6e). The addition of IL-17 antibodies blocked the upregulation of these genes (Supplementary Fig. 6e). In a primary OA model, which mimicked only trauma-induced joint destabilization but without IA blood treatment (DMM model), the OARSI score (cartilage damage) was similar with hemarthrosis model for a longer time after surgery (12 weeks) (Supplementary Fig. 8a, b). However, the serological COL II content did not reach the level as the hemarthrosis model (Supplementary Fig. 8f). Neither the activation of CD4^+^ T cells nor the liver impairment were observed in the DMM model (Supplementary Fig. 8c–e, g). In all, these data suggested that blood induces the sCOL II release both in vivo and in vitro, which consequently activates CD4^+^ T cells and leads to abnormal liver functions.

### Cartilage Akt signaling is involved in hemarthrosis-induced damage

**Fig. 5:**
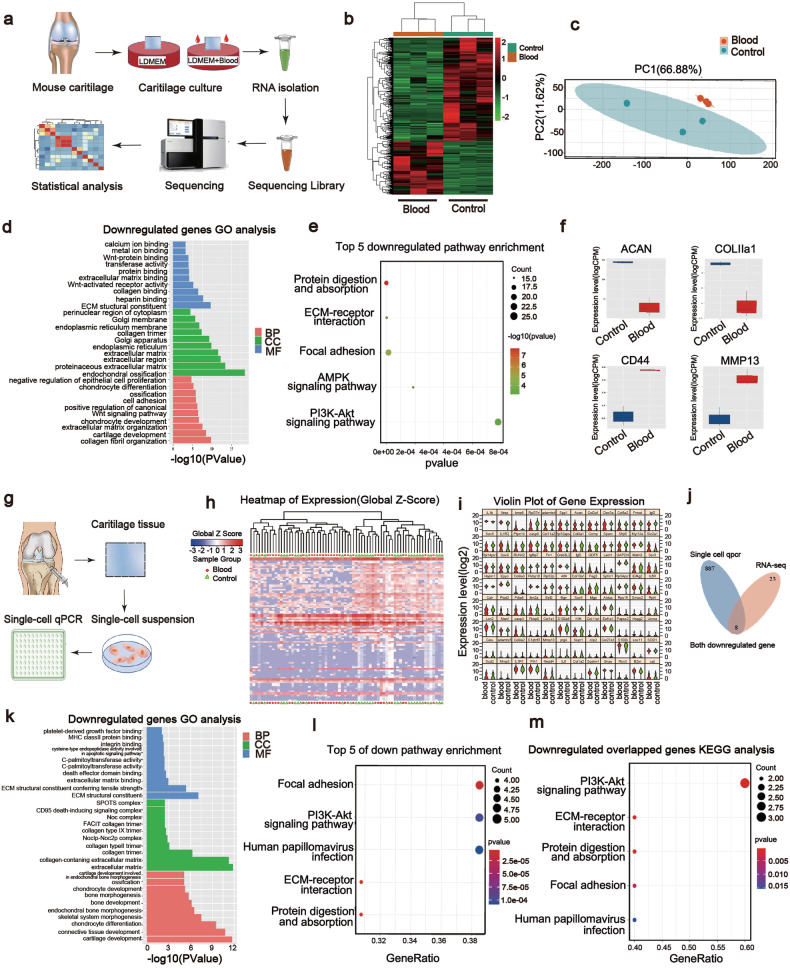
Transcriptomic analysis reveals the downregulation of cartilage Akt signaling pathway by blood. **a** The procedure of RNA-Seq analysis of mice cartilage explants. **b** Heatmap of the RNA-seq data (*n* = 3). **c** Principal component analysis (PCA) of the RNA-seq data (*n* = 3). **d** GO (Gene Ontology) analysis for biological pathway (BP), cellular components (CC) and molecular function (MF) of downregulated genes in Blood group from RNA-Seq data (*n* = 3). **e** KEGG pathway analysis of downregulated pathways in Blood group from RNA-seq data (*n* = 3). **f** Representative chondrogenic markers (*Acan*, *Col IIa1*, and *Cd44*) and destructive marker (*Mmp13*) expression from RNA-seq data (*n* = 3). **g** The procedure of single-cell qPCR of mice cartilage explants. **h** Heatmap showing the hierarchical clustering (HC) analysis including all the cells in single-cell qPCR. The orders of relative genes from top to bottom are the same as Supplementary Table 5. **i** Violin plot showing the expression of each single gene in single-cell qPCR data. **j** The overlapping of downregulated genes of Blood group from both RNA-seq and single-cell qPCR data. **k** GO analysis of downregulated genes in Blood group from single-cell qPCR data. **l** KEGG pathway analysis of downregulated genes in Blood group from single-cell qPCR data. **m** KEGG pathway analysis of overlapped downregulated genes in Blood group. In single-cell qPCR analysis, 36 blood-treated cells and 34 control cells were measured

Since cartilage-derived components are, at least in part, responsible for the pathological joint–liver axis, we next aim to clarify the key genes and signaling pathways involved in blood-induced cartilage injury. Therefore, RNA-seq and single-cell qPCR analysis were utilized for detecting blood-treated cartilage explants (Fig. 5a, g). Heatmap and volcano plot from RNA-seq showed 461 upregulated genes and 887 downregulated genes in Blood group (Fig. 5b and Supplementary Fig. 9a). Then, the representative differentially expressed genes (DEGs) in RNA-seq together with other specific-marker genes from previous studies (Supplementary Table 5, 96 genes in total) were selected to perform single-cell qPCR (Fig. 5g). Principal component analysis (PCA) of RNA-seq and hierarchical clustering (HC) analysis of single-cell qPCR revealed that the samples and cells from the two groups could be separated into two major clusters (Fig. 5c, h). The cells from Blood group were mainly concentrated in the left population (Fig. 5h). Meanwhile, cells from Blood group displayed relatively lower expression levels of chondrogenic genes such as *Col2a1* and *Acan* while displayed higher expression levels of several cartilage degradation-related genes in single-cell qPCR and RNA-seq data (Fig. 5i, f). GO analysis of the RNA-seq data revealed that chondrogenic terms such as cartilage development, collagen fibril organization and extracellular matrix organization were downregulated in Blood group (Fig. 5d), which were similar to the results from single-cell qPCR data (Fig. 5k). KEGG pathway analysis for RNA-seq data revealed that ECM–receptor interaction, PI3K-Akt signaling pathway and AMPK signaling pathway were significantly downregulated in Blood group compared with Control group (Fig. 5e), which were also similar to those from single-cell qPCR data (Fig. 5l). The terms of GO and KEGG pathway analysis of upregulated genes from RNA-seq and single-cell qPCR were not very consistent although some terms related to inflammation and rheumatoid arthritis could be found (Supplementary Fig. 9b–e). Therefore, we integrated the downregulated genes from these 2 experiments, which revealed 8 overlapping genes (Fig. 5j and Supplementary Fig. 9f). KEGG pathway analysis of the genes showed that the PI3K-Akt signaling pathway was the most significantly downregulated one (Fig. 5m). Collectively, bulk and single-cell RNA analysis of the blood-treated cartilage explant revealed several decreased chondrogenic terms in GO analysis and the most significantly downregulated PI3K-Akt signaling pathway in KEGG analysis.

### Akt activator rescues cartilage degradation

**Fig. 6:**
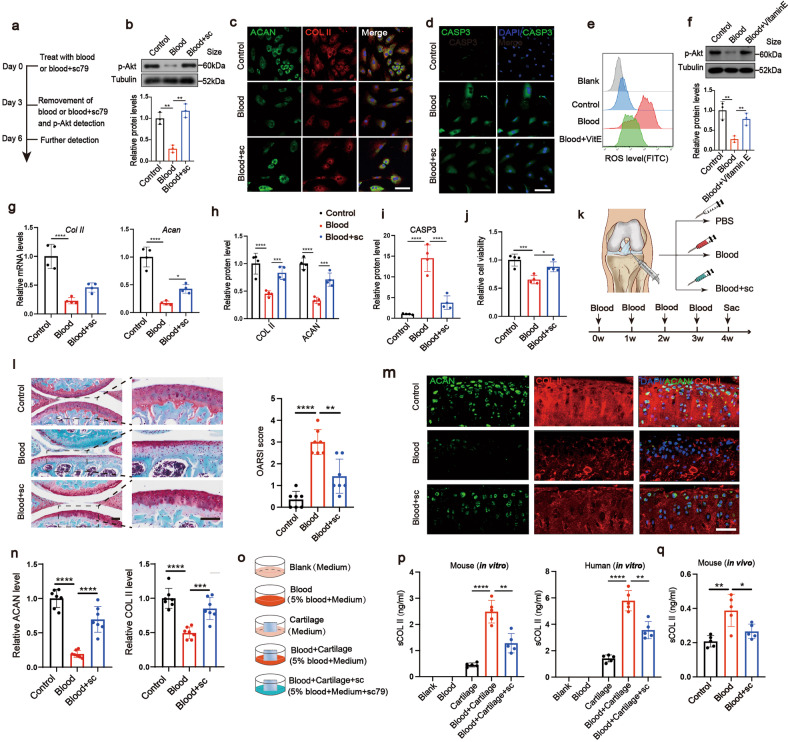
Akt activator rescues cartilage degradation. **a** The experimental procedure for re-activating Akt in vitro. **b** Western blot analysis of p-Akt expression and quantification normalized by Tubulin (*n* = 3). **c**, **d** Immunofluorescence analysis of ACAN (green), COL II (red), C-caspase-3 (green) in the chondrocytes (scale bar, 100 µm). **e** ROS (FITC) level analysis with or without VitminE (VitE) scavenge. **f** Western blot analysis of p-Akt expression and quantification normalized by Tubulin on chondrocytes (*n* = 3). **g** The chondrocyte mRNA expression of *Acan* and *Col II* in each group was measured by qPCR (*n* = 4). **h**, **i** The quantitation of ACAN, COL II, C-caspase-3 protein level from (**c**, **d**), respectively (*n* = 4). **j** Chondrocyte viability by CCK-8 assay (*n* = 4). **k** The experimental procedure for re-activating Akt in vivo. **l** SO staining (scale bar, 100 µm) and corresponding OARSI quantification (*n* = 7). **m**, **n** Immunofluorescence analysis of ACAN (green) and COL II (red) in the collected knee joints treated with blood (scale bar, 65 µm) and the respective quantitation (*n* = 7). **o** The experimental procedure for sCOL II detection in vitro. In total, 5 μg/ml sc79 was added into the medium. **p** sCOL II level in the supernatant of treated human and mice cartilage explants by ELISA assay (*n* = 5). **q** Serum COL II level of mice in vivo (*n* = 5). *P < 0.05, **P < 0.01, ***P < 0.001, ****P < 0.0001

As the activator of ECM–receptor interaction pathway only showed mild protective effects between the activators of the top 2 pathways derived from our transcriptomic data (Supplementary Fig. 10), the function of the phosphorylation activator of the Akt, sc79^23^ was explored in the next experiments (Fig. 6a, b). The blood-induced decrease of cell viability and *Bcl-2* expression and the increase of CASP3 and *Bax* expression were significantly reversed in the Blood+sc group (treated with blood containing sc79) (Fig. 6d, i, j and Supplementary Fig. 11a), indicating that sc79 reversed blood-induced chondrocyte apoptosis. The decreased ACAN and COL II expression, at both mRNA and protein levels, as well as the increased *Mmp-3* and *Mmp-13* expression in the Blood group were all alleviated by sc79 (Fig. 6c, g, h and Supplementary Fig. 11b). To further confirm the roles of Akt pathway, we first found that one of the upstream events of the Akt is elevated ROS because reversing the elevated ROS in Blood group by vitamin E (VitE) significantly reversed the downregulation of p-Akt (Fig. 6e, f). We then found that VitE could protect against the decreases of cell viability and loss of chondrocyte phenotypes by blood treatment (Supplementary Fig. 12b, c). The use of Akt inhibitor, Ly294002 (ly) (Supplementary Fig. 12a), caused severer decreases of cell viability and loss of chondrocyte phenotypes compared with blood treatment alone (Supplementary Fig. 12b, c). On the other hand, our RNA-seq data has found out several genes related to the Akt activation (Supplementary Fig. 9f). *Igf2* is the most recognized one among them,^24^ thus we also explored the roles of *Igf2* in Akt activation and chondrocyte phenotype. We firstly confirmed that the blood significantly downregulated *Igf2* expression (Supplementary Fig. 13a). Then, we added Igf2 into the medium of blood-treated chondrocytes. We found that Igf2 significantly reversed blood-induced p-Akt downregulation (Supplementary Fig. 13b). Igf2 also reversed blood-induced downregulation of cell viability and the level of chondrogenic markers of chondrocytes (Supplementary Fig. 13c, d). For in vivo experiments (Fig. 6k), after IA sc79 treatment, the damage of articular cartilage, including ECM degradation and attenuation of cartilage thickness, was less severe in Blood+sc group compared with the Blood group (Fig. 6l). The protein contents of ACAN and COL II of articular cartilage were significantly improved in the Blood+sc group (Fig. 6m, n). Having confirmed that sc79 attenuates cartilage damage, we then explored whether it could alleviate COL II release. For in vitro experiments (Fig. 6o) of mouse and human samples, the Blood+Cartilage+sc group significantly decreased the level of sCOL II compared with Blood+Cartilage group (Fig. 6p). For in vivo experiments, serological COL II concentration was suppressed in Blood+sc group compared with Blood group (Fig. 6q). Taken together, we found that treatment of cartilage with Akt activator reverses the blood-induced cartilage damage and COL II release both in vivo and in vitro.

### Intra-articular injection of Akt activator rescues systemic changes in mouse and pig model

**Fig. 7:**
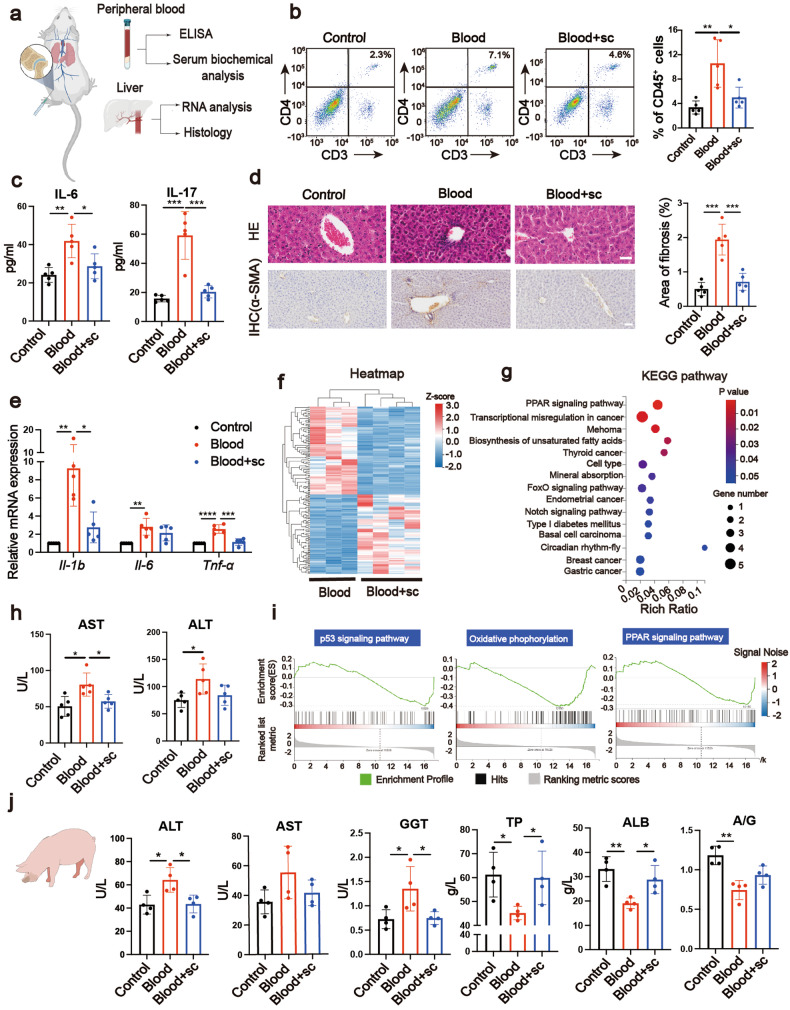
Intra-articular injection of Akt activator rescues systemic changes in mouse and pig model. **a** The evaluation procedure of mice liver and blood. **b** Flow cytometric measurement of the percentages of CD4^+^ T cells in peripheral blood, gated on CD45^+^ (*n* = 5). **c** Serum IL-6 and IL-17 levels detected by ELISA (*n* = 5). **d** HE staining (scale bar, 40 µm), IHC analysis (scale bar, 50 µm) on α-SMA protein and corresponding quantification of fibrosis areas of the collected liver (*n* = 5). **e** The liver mRNA expression of *Il-1b*, *Il-6*, and *Tnf-α* measured by qPCR (*n* = 5). **f** Heatmap of liver tissue from RNA-seq data (*n* = 3). **g** KEGG pathway analysis of downregulated genes in Blood+sc group of liver tissue (*n* = 3). **h** Serum biochemical analysis of AST and ALT (*n* = 5). **i** GSEA results of the RNA-seq data (*n* = 3). **j** The serological markers of liver function of pigs including ALT, AST, GGT, TP, ALB, and A/G (*n* = 4)

Since re-activation of Akt reverses local cartilage damage and COL II release, we then investigated whether blocking cartilage damage by IA intervention of Akt could provide systemic recoveries (Fig. 7a). To exclude the effects of IA sc79 treatment on extra-articular tissue in the pathological joint–liver axis, we revealed that the p-Akt level of PBMCs and liver tissue were unchanged after IA sc79 treatment (Supplementary Fig. 14). The increased levels of peripheral CD4^+^ T cells and serological IL-6 and IL-17 in Blood group were significantly suppressed in Blood+sc group (Fig. 7b, c). As for the liver, less immune cell infiltration, significant recovery of liver inflammation markers (*Il-1b*, *Il-6*, and *Tnf-α*) and significant recovery of serological ALT and AST were observed in Blood+sc group compared with the Blood group (Fig. 7d, e, h). Liver fibrosis areas were also significantly recovered in Blood+sc79 group (Fig. 7d). To further confirm the key roles of blood-treated cartilage tissue causing peripheral damage, the Blood+sc79 group was further intravenously injected conditioned media from blood-treated cartilage explant (with or without the addition of sc79 into the media) and blood-treated fibroblasts (Supplementary Fig. 15a). We found that, though IA application of Akt activator kept the ALT, AST, and IL-17 level at normal level, conditioned media from blood-treated cartilage explant increased the level of ALT, AST, and IL-17 again; conditioned media from Blood+sc79 treated cartilage explant reversed the upregulation of ALT, AST, and IL-17 level; conditioned media from blood-treated fibroblasts did not show a significant difference in ALT, AST, and IL-17 level compared with control conditioned media (Supplementary Fig. 15b, c). In concert with Fig. 2m–o, we performed RNA-seq analysis for the liver in Blood group and Blood+sc group. The heatmap showed the 86 upregulated genes and 95 downregulated genes of Blood+sc group (Fig. 7f). Next, the results of KEGG pathway analysis and GSEA analysis showed that some upregulated pathways in Blood group compared with Control group (Fig. 2n, o) were downregulated in Blood+sc group, mainly related to steatosis, fat metabolism and apoptosis, such as PPAR signaling pathway, biosynthesis of unsaturated fatty acid, oxidative phosphorylation and p53 signaling pathway (Fig. 7g, i), suggesting the recovery effects in Blood+sc group. We finally performed IA injection of PBS, Blood or Blood+sc in pig model. Similar with our clinical data, increased level of serological AST, ALT, and GGT and decreased level of TP, ALB, and A/G were found in Blood group compared with Control group (Fig. 7j). All these indexes were rescued, at least in part, in Blood+sc group (Fig. 7j). Taken together, the systemic activation of CD4^+^ T cells and liver damage were significantly reversed after prevention of the cartilage damage and COL II release by IA application of Akt activator.

## Discussion

The systemic and remote organ responses induced by the knee joint have not been investigated, and the changes toward cartilage-relevant signal pathway during hemarthrosis are still poorly understood. The main findings of this study are as follows. First, liver impairment can be induced by post-traumatic joint damage in humans and IA blood in mice. Next, the pathological joint–liver axis is attributed to cartilage damage-induced upregulation of serological COL II constituents and the sCOL II-activated CD4^+^ T cells. Finally, the blood induces cartilage damage by inhibition of cartilage Akt pathway and IA application of Akt activator recovers systemic changes caused by hemarthrosis.

First, we revealed a pathological joint–liver axis in both mice and humans. Previous studies mainly focused on the communication between the organs with active metabolic functions, large area (volume) and/or important life-support functions.^11,25^ Our study demonstrated that hemarthrosis can cause remote liver responses, suggesting that structurally closed and even avascular and aneural cartilaginous tissue can also communicate with distant organs, for which previous studies have only focused on the communications among intra-articular tissues.^2^ As hemarthrosis is a clinically common disease involved in traumatic knee injuries, joint surgery, and hemophilia patients,^2,4,12^ this finding is important for public liver health, and the liver functions of these patients should also be concerned. Our clinical data showed that primary OA did not cause a higher chance of liver abnormalities, which is in consistent with our chronic OA (DMM) mouse model that fails to cause liver damage. This may be because hemarthrosis mice model or post-traumatic joint damage is a kind of relatively acute injury and causes a sudden release of high level of sCOL II, which could activate immune responses. Relatively chronic and mild arthropathies are not easily to cause systemic responses. However, detective serum markers have been reported in other types of OA,^13^ providing the opportunity to cause systemic responses. We also noted that, in clinical data, the liver damage in PTJD group was not very strong and existing high within-group variation. As other arthropathies such as primary OA can be further classified,^26^ which subcluster of PTJD patients tends to exhibit liver impairment can also be further identified. Although liver damage was found during hemarthrosis, the influence of other organs except the spleen were not observed at the same time point (data are not shown). Our previous study found that, after abdominal wall implantation of biomaterials (silk fibroin and polypropylene), liver impairment is the most significant among different organ defects, including kidney, heart, spleen etc.^10^ These two studies together suggested that liver is a relatively fragile organ that is sensitive to systemic immune-metabolic alteration.

Next, we found that the matrikine-activated CD4^+^ T cells mediate the joint–liver axis. CD4^+^ T cells have been reported to mediate several organ communications, including gastrointestinal infection-triggered joint disease,^27^ food-allergic enteropathy-triggered bone damage,^28^ and AKI-triggered pulmonary inflammation.^29^ We found, for the first time, that the CD4^+^ T cell can be activated during hemarthrosis. Studies have widely explored that abnormal CD4^+^ T cell activation, mainly including Th1 and Th17 cells, can contribute to liver inflammation and other diseases.^21^ Our findings are consistent with previous reports that the Th17 cells are activated and IL-17 are upregulated, while the protective Treg cells are inhibited. Our findings further enriched the immunological functions of CD4^+^ T cells and the imbalance of Th17/Treg cells in organ communications and liver pathology. In addition, our results suggested that although cartilage lacks blood vessels, nerves, and lymphatic tissue, it can communicate with other distant tissues and/or cells by releasing ECM into the peripheral blood. Similar to other autologous components,^19,20^ sCOL II can directly activate CD4^+^ T cells in humans with rheumatic disease and to a lesser extend in healthy control.^30^ In C57BL/6 mice, combined application of heterologous sCOL II and Freund’s adjuvant activated T cells and caused rheumatoid arthritis.^31^ In our study, CD4^+^ T cells exhibited tolerance to normal level of autologous sCOL II, while they were activated when the sCOL II produced by cartilage degeneration was released into peripheral blood. These data suggested that sCOL II-produced cartilage degradation can be an alarming molecule like other soluble matrix-degradation fragments to cause inflammation.^11,17,32^ Besides, our study provides direct evidence the sCOL II leads to imbalance of Th17/Treg, similar to the pathological condition of rheumatoid disease.^30^ The release of autologous substance in relatively close parts of the body, with which the immune cells cannot come in contact in the physiological state, can trigger autoimmune responses, such as the exposure of retinal and sperm antigen.^33,34^ Our results are consistent with and extend this theory by revealing the roles of joint-derived sCOL II. We noted that joint injury would secret various factors not only COL II,^12,13^ and liver injury also alters many metabolites such as cholesterol.^25^ Whether there are other factors besides sCOL II and CD4^+^ T cells to regulate the joint–liver axis can be further explored.

Finally, we focused on the starting point of the joint–liver axis and found that the Akt pathway mediates blood-induced cartilage damage. Blood in the cartilage microenvironment can directly induce chondrocyte apoptosis.^7–9^ Similarly, our in vitro study demonstrated that blood downregulates cell viability and upregulates apoptosis in a dose-dependent manner. Since prevention of apoptosis can alleviate blood-induced cartilage degeneration,^9^ the cellular mechanism of the apoptosis-promoting effect of blood on cartilage needs to be understood. Studies have shown that the Akt signaling pathway has dual functions. On the one hand, previous studies have shown that the Akt signaling pathway of chondrocytes is downregulated when exposed to IL-1b and TNF-α, leading to cell apoptosis,^35,36^ while activating the Akt signaling pathway by FGF18 can alleviate OA progress.^36^ Our study found that blood downregulates the Akt signaling pathway while activation of the pathway alleviates blood-induced cartilage damage both in vivo and in vitro, which is the first study to confirm the role of Akt in hemarthrosis. Previous study found that the p-Akt can interact with Bcl-2 family protein to resist apoptosis,^37^ which may share a similar downstream mechanism with our study. On the other hand, prolonged activation of Akt signaling was reported to cause accumulation of reactive oxygen species (ROS) and trigger senescence of chondrocyte and OA.^38^ The reasons for the difference could be that the Akt signaling is disruptive both at lower and sustained higher than normal level. Besides, joint damage for different reasons may exhibited different molecular markers and mechanism.^26^ Therefore, maintaining the Akt signaling pathway in a normal range is important for cartilage homeostasis.

## Materials and methods

### Animal experiments

Animal experiments were conducted with the approval of the Zhejiang University Experimental Animal Welfare and Ethics Committee under Institutional Animal Care and Use Committee guidelines (ZJU20230014 for mouse model, ZJU20230341 for pig model). Previous hemarthrosis mice model, which mainly focused on local joint damage, was established by whole-body deletion of the coagulation factor together with joint puncture.^39^ The hemophilic model would cause systemic changes. To investigate the hemarthrosis-caused liver responses in normal organism, we referenced IA injection model previously used for large animal.^40^ Though single bleeding would damage the joint function in human,^41^ mice that only received three or four doses of IA blood injection exhibited significant decrease of sport ability (Supplementary Fig. 16). The serological markers of liver dysfunction were increased after three or four doses of blood injection and the group treated with four doses of blood showed significant increase compared with control (Supplementary Fig. 3A). Therefore, to imitate clinical phenomenon (worse serological liver indexes), the knee hemarthrosis model was established on 10-week-old mice by 4-time blood injection (8 μl per leg) into the bilateral knee articular cavity, once a week. The blood injected was autologous. After collecting the blood through cutting a small area at the terminal of tail, the blood was collected and immediately injected without removing or adding any factors. PBS was used in the Control group. Blood containing 25 μg/ml sc79 (S7863, Selleck, USA) was used in Blood+sc group. The C57BL/6 mice were sacrificed 1 week after final injection, and the joints, the liver, the spleen, and the peripheral blood were collected for subsequent studies. The hemarthrosis model was also established on 6-month-old Yorkshire pigs by 4-week blood injection (2 ml per leg) into the two knee articular cavities, twice a week. PBS was used in the Control group and blood containing 25 μg/ml sc79 was used in Blood+sc group. The Yorkshire pigs were sacrificed 1 week after final injection, and the peripheral blood were collected for subsequent studies. Mouse DMM model was referenced by previous study.^42^

For systemic COL II antibody (ab34712, Abcam, UK) treatment to block the elevated serological COL II, the antibody were intraperitoneal injection at a dose of 3 mg/kg mice. According to the change of serological COL II level, for the first and second blood injection, the antibody were injected once a week, 1 h after blood treatment. For the third and fourth blood injection, the antibody were injected twice a week, 1 h and 3 d after blood injection. IgG (I5006, Sigma, Germany) was used as a control. For IL-17 antibody (clone 17F3, BioXCell, USA) treatment, the antibody was intraperitoneal injection with 300 μg/mice an hour after very blood treatment at the 1st and 2nd IA blood injection, every 3 d after the 3rd IA blood injection. For the intravenous injection of conditioned media, the frequency is same as antibodies injection, 40 μL/injection. The fibroblasts used in this study were L929 cells from ATCC. The conditioned media were centrifugated to exlude cells before injection.

### Clinical data

The data of clinical serum biochemical test of the study were collected with the approval of Second Affiliated Hospital of Zhejiang University School of Medicine (20230735). Baseline characteristics is listed as Supplementary Table 1. During the process of data collection of PTJD group, patients with trauma-induced muti-organ damage were excluded, while those only diagnosed with joint damage were collected. Patients with drug, surgery and diseases history that may influence liver functions were also excluded.

### Chondrocyte isolation and cell experiments

Mice chondrocytes were isolated from the knee cartilage of postnatal mice (day 0–4) following a previously published method.^43^ The collagenaseII-digested cells were cultured with DMEM/F12 medium (Gibco, USA) with 10% fetal bovine serum and 1% penicillin/streptomycin (P/S, Gibco, USA) at 37 °C with 5% CO_2_. For the detection of ROS level in chondrocytes, the DCFH-DA (S0033S, Beyotime, China) was used for flow cytometry detection according to the manufacturer’s instructions. Hepatocytes used in this study were AML12 cells from ATCC. The cells were cultured and treated with serum (10%, v/v) from the Control or Blood group for 24 h before detection, referenced by the previous study.^25^ For the activation of splenocytes and PBMCs in vitro, 50 μg/ml (terminal concentration) mouse sCOL II (MC22, Elastin Products Company, USA) was directly added into the media of the cells, which were derived from the hemarthrosis model. After 24-h co-culture, the cells and supernatant were detected or used for the treatment of hepatocytes. 15 μg/ml (terminal concentration) of IL-17 antibody or IgG control was used for in vitro study. For in vitro IGF-2 treatment, 10 ng/ml IGF-2 (292-G2, R&D Systems, USA) was used.

For in vitro blood treatment, the transwell system was used to deal with the cells during blood (5% or 10%, v/v) or blood (5%) containing 5 μg/ml sc79 treatment. Ly294002 (ly) (20 μM, S1105, Selleck, USA) and VitE (100 μM, S4686, Selleck, USA) and pyrintegrin (2 or 10 μM, E0462, Selleck, USA) were also used to treat chondrocytes together with blood (5%).The whole blood was added into the upper well and the chondrocytes were cultured in the lower well. The blood was removed after three days of treatment and the chondrocytes were cultured for another 3 days before collection. Cell viability was assayed by CCK-8 kit (CK04, DOJINDO, Japan) according to the manufacturer’s instruction. For treatment of cartilage explant, 5% (v/v) blood, blood+sc (5 μg/ml) or PBS was directly added into culture medium and cultured for 3 days before detection.

### Micro-CT analysis

The fixed joint samples were scanned using micro-CT (U-CT-XUHR, MILabs, Netherlands) at 4 μm resolution. The three-dimensional (3D) structures of the calvarium were reconstructed through MILabs-Rec, and analyzed by IMALYTICS Preclinical 2.1. software of the micro-CT.

### Von Frey test

The von Frey test was performed mimicking a previous study.^44^ The package of the experiment was bought from Stoelting, USA. Briefly, allodynia was evaluated by applying von Frey hairs in ascending order of bending force (force range: 0.02, 0.04, 0.07, 0.16, 0.40, 0.60, 1.0, 1.4 or 2.0 g). The von Frey hair was applied perpendicular to the plantar surface of the hind paw (avoiding the toe pads) for 2–3 s. If no response, the next higher strength of hair was used. If a withdrawal response occurred, the paw was re-tested, starting with the next descending von Frey hair until no response occurred. Four more measurements were made after the first difference was observed. The 50% PWT was determined by using the following formula:

$$50 \% {\rm{PWT}}=({10}^{{\rm{Xf}}+\kappa \delta })/10,000,$$

where Xf is the exact value (in log units) of the final test of von Frey hair, κ is the tabular value for the pattern of the last six positive/negative responses, and δ is the mean difference (in log units) between stimuli. The threshold force required to elicit paw withdrawal (median 50% withdrawal) was determined twice on each hind paw (and averaged) on each testing day, with sequential measurements separated by at least 10 min.

### Histology, immunohistochemistry (IHC), and immunofluorescence (IF) assays

Tissue specimens were fixed in 4% paraformaldehyde, washed with running water, dehydrated in a graded ethanol series, vitrified with dimethylbenzene, and embedded in paraffin. Paraffin sections (7 μm) were deparaffinized in xylene, hydrated with gradient ethanol, and stained with standard hematoxylin and eosin (HE) and safranin O/Fast green staining (SO) staining procedures. Cartilage histological scores were calculated from the results of the staining according to the OARSI atlas.^45^

For IHC or IF analysis, sections were incubated at 4 °C overnight with primary antibodies (Supplementary Table 3). For IHC, the sections were then incubated with horseradish peroxidase-linked secondary antibodies (Supplementary Table 3) for 1 h, and the staining was visualized with diaminobenzidine (DAB) solution (DA1010, Solarbio, China). For IF, sections or cells were subsequently incubated with fluorescein-conjugated secondary antibodies for 1 h and observed under a confocal fluorescence microscope (Olympus, Japan).

### Treadmill running

To assess the exercise performance of the mice, they were subjected to treadmill activity at an initial speed (1 m/min) and a gradually increasing speed (2 m/min^2^). An electric foot shock was used to stimulate mice to run. The number of electric foot shocks was calculated, and the test was terminated when the maximum shock count reached 350.

### RNA-seq

The cartilage samples from the newborn mice were collected in the dishes and dealt with blood. The liver samples were collected from the joint-bleeding mice model. RNAseq and library construction was performed by the Beijing Genomics Institution (BGI, China). Briefly, total RNA was extracted from the tissues using Trizol (Invitrogen, USA) according to manual instructions. About 60 mg of tissues were ground into powder by liquid nitrogen in a 2-mL tube for RNA extraction. Then, 25–100 μL of DEPC-treated water was added to dissolve the RNA. Total RNA was qualified and quantified using a Nano Drop and Agilent 2100 bioanalyzer (Thermo Fisher Scientific, USA). Next, purified mRNA was fragmented with fragment buffer at appropriate temperature. First-strand cDNA was generated using random hexamer-primed reverse transcription, followed by a second-strand cDNA synthesis. The cDNA fragments obtained from the previous step were amplified by PCR, and products were purified by Ampure XP Beads. The final library was amplified with phi29 to make DNA nanoball (DNB, China) which had more than 300 copies of one molecular, DNBs were loaded into the patterned nanoarray and single end (liver tissue) or double end (cartilage tissue) 50 bases reads were generated on BGIseq500 platform (BGI-Shenzhen, China).

### Quantitative real-time PCR

Quantitative real-time PCR (qPCR) was conducted to evaluate the mRNA levels of genes. At the endpoint of each experiment, the cells or tissues were washed 2 times in PBS and then added with Trizol reagent (9109, TaKaRa, Japan) for RNA extraction. Reverse transcription was conducted by a commercial kit (R323, Vazyme, China) according to the manufacturer’s instructions. The primers used for the qPCR analysis in this study are listed in Supplementary Table 2. qPCR reactions were performed using the TB Green PCR kit (Q711, Vazyme, China) according to the manufacturer’s instructions.

### Western blot

RIPA lysis buffer containing protease and phosphatase inhibitors (78442, ThermoFisher Scientific Inc, USA) were used for cell lysis, BCA assay (P0012S, Beyotime, China) was used for quantification of the protein concentration. Overall, 25 μg total proteins were loaded. The gels were run for an hour and half, and the separated proteins were subsequently transferred to PVDF membranes. The membranes were blocked for an hour and then incubated with primary antibodies (Supplementary Table 3) at 4 °C overnight. After they were incubated with secondary antibodies (Supplementary Table 3) at room temperature for 1 h, the blot was imaged (Clinx Science Instruments, China). For detection of p-STAT3 protein, after treatment of sCOL II to mixed splenocytes, CD4^+^ T cells were sorted and detected by western blot.

### Flow cytometry analysis

For flow cytometry analysis of blood and spleen sample, followed by lysis of red blood cells, more than 1 × 10^5^ cells were suspended in 100 μl PBS and incubated with fluorescence-conjugated primary antibody (Supplementary Table 3) on ice for 1 h. The cells were then washed and suspended in PBS and was detected using a CytoFLEX S flow cytometer (Beckman Coulter, USA). FlowJo software (BD Biosciences, USA) was used to analyze the data.

### Blood analysis

The cytokines in mouse serum were detected using ELISA assay kits (for IL-6, ml063159, Mlbio, China) (for IL-17, DY421, R&D system, USA) according to the manufacturer’s instructions. Mice (ml063001, Mlbio, China) and human (ml057473, Mlbio, China) sCOL II was detected using ELISA assay kit. The serum biochemical markers were detected by an automatic biomedical analyzer (BS-240vet, Mindray, China).

### Single-cell mass cytometry

CyTOF (PLT Tech., China) staining was performed following a previously published method.^46^ Cells were washed with 1× PBS once and then stained with 100 μL of 250 nM cisplatin (Fluidigm, USA) for 5 min on ice to exclude dead cells, and then incubated in Fc receptor blocking solution before stained with surface antibodies cocktail for 30 min on ice. Cells were washed with FACS buffer (1xPBS + 0.5%BSA) for twice and fixed in 200 μL of intercalation solution (Maxpar Fix and Perm Buffer containing 250 nM 191/193Ir, Fluidigm, USA) overnight. After fixation, cells were washed once with FACS buffer and then perm buffer (eBioscience, USA), and stained with antibodies cocktail (Supplementary Table 4) for 30 min on ice. Cells were washed and resuspended with deionized water, added into 20% EQ beads (Fluidigm, USA), and acquired on a mass cytometer (Helios, Fluidigm, USA).

Cell clusters were identified based on marker expression distribution according to standard definitions of cell type: CD4^+^ T cells (B220^−^ Gr1^−^ CD3e^+^ TCRb^+^ CD4^+^ CD8^−^), CD8^+^ T cells (B220^−^ Gr1^−^ CD3e^+^ TCRb^+^ CD4^−^ CD8^+^), gdT (B220^−^ Gr1^−^ CD3e^+^ TCRb^−^ TCRgd^+^), NK (NK1.1^+^ CD3e^−^ B220^−^ CD19^−^), B cells (CD3e^-^ B220^+^ CD19^+^), monocyte (CD11b^+^ Ly6C^+^ NK1.1^−^ CD3^-^ CD19^−^ B220^−^ FcerIa^−^ siglecF^−^ Ly6G^−^), DC (CD3e^−^ B220^+^ CD19^-^ CD317^+^), Neutrophil (CD11b^+^ Ly6G^+^), eosinophil (CD11b^+^ SiglecF^+^), basophils (CD11b^+^ Fcer1a^+^ SiglecF^−^ Ly6G^−^).

### Single-cell real-time quantitative PCR and analysis

Single-Cell Real-Time Quantitative PCR (Single-cell qPCR) was performed according to standard protocols provided by Fluidigm (USA). Briefly, cartilage samples from the joint-bleeding mice model were digested and suspended. Single cells were captured by micropipette under a 40x microscope. Amplified complementary DNA libraries were generated using the C1 Single-Cell Auto Prep Module 1 and Module 2 kits (Fluidigm, USA) and a mixture of outer primers specific to 96 genes (Supplementary Table 5). Genes were selected on the basis of established markers for cell adhesion, inflammation, and chondrocyte lineage based on the scientific literature. Single-cell QPCR analysis were performed with inner (nested) primers on the BioMark platform (Fluidigm, USA) using 96 Dynamic Array IFC chips (Fluidigm, USA), according to the manufacturer’s instructions. Single-cell qPCR data with Ct values were analyzed by the SINGuLAR Analysis Toolset 3.6.2 software (Fluidigm, USA).

### CD4^+^ T-cell isolation and transfer to Rag1-deficient mice

Spleens from C57BL/6 mice were used to isolate CD4^+^ T cells by isolating CD45^+^CD3^+^CD4^+^CD19^-^CD11b^-^NK1.1^-^ cells using a Beckman moflo Astrios EQ flow sortor. Cells were washed and resuspended in saline for intravenous injection. In all, 2.5 × 10^6^ sorted cells were injected to Rag1-deficient mice (GemPharmatech, China) 3 days prior to blood injection.

### Data analysis

All data were shown as the mean ± SE. Statistical analysis was performed and charts were constructed with GraphPad Prism version 8.0. Statistical comparisons were calculated using a two-tailed Student’s *t* test (2 groups) or one-way ANOVA with Tukey’s correction (multiple groups). A *P* value of <0.05 was considered statistically significant. In transcriptomic data, *P* value of <0.05 and fold change ≥2 were considered as DEGs.

### Supplementary information

Supplementary materials

## Data Availability

The RNA-sequencing data have been deposited in the China National Center for Bioinformation (CNCB) under GSA accession numbers (CRA015112). Cytof data have been deposited in CNCB under OMIX accession numbers (OMIX005885). All data in this article are available upon reasonable request from the corresponding authors.

## References

[1] Thomas AC, Hubbard-Turner T, Wikstrom EA, Palmieri-Smith RM (2017). Epidemiology of posttraumatic osteoarthritis. J. Athl. Train..

[2] Lyons LP, Weinberg JB, Wittstein JR, McNulty AL (2021). Blood in the joint: effects of hemarthrosis on meniscus health and repair techniques. Osteoarthr. Cartil..

[3] Gualtierotti R, Solimeno LP, Peyvandi F (2021). Hemophilic arthropathy: current knowledge and future perspectives. J. Thromb. Haemost..

[4] Ravi B, Hosack L, Backstein D, Spangehl M (2019). Recurrent hemarthrosis after total knee arthroplasty: evaluation and treatment. J. Am. Acad. Orthop. Surg..

[5] Roosendaal G (1998). Iron deposits and catabolic properties of synovial tissue from patients with haemophilia. J. Bone Jt. Surg. Br..

[6] Tajima T (2017). Hemoglobin stimulates the expression of ADAMTS-5 and ADAMTS-9 by synovial cells: a possible cause of articular cartilage damage after intra-articular hemorrhage. BMC Musculoskelet. Disord..

[7] van Vulpen LF (2015). IL-1beta, in contrast to TNFalpha, is pivotal in blood-induced cartilage damage and is a potential target for therapy. Blood.

[8] Pan Z (2022). Naringenin protects against iron overload-induced osteoarthritis by suppressing oxidative stress. Phytomedicine.

[9] Hooiveld M (2003). Short-term exposure of cartilage to blood results in chondrocyte apoptosis. Am. J. Pathol..

[10] Peng Z (2023). Biomaterial based implants caused remote liver fatty deposition through activated blood-derived macrophages. Biomaterials.

[11] Dokoshi T (2021). Skin inflammation activates intestinal stromal fibroblasts and promotes colitis. J. Clin. Investig..

[12] Pasta G (2020). The progression of hemophilic arthropathy: the role of biomarkers. Int J. Mol. Sci..

[13] Kumavat R (2021). Biomarkers of Joint damage in osteoarthritis: current status and future directions. Mediators Inflamm..

[14] Romier B (2018). Production of elastin-derived peptides contributes to the development of nonalcoholic steatohepatitis. Diabetes.

[15] Akthar S (2015). Matrikines are key regulators in modulating the amplitude of lung inflammation in acute pulmonary infection. Nat. Commun..

[16] Masood A (2015). Neutrophil elastase-induced elastin degradation mediates macrophage influx and lung injury in 60% O_2_-exposed neonatal rats. Am. J. Physiol. Lung Cell Mol. Physiol..

[17] Goodall KJ, Poon IK, Phipps S, Hulett MD (2014). Soluble heparan sulfate fragments generated by heparanase trigger the release of pro-inflammatory cytokines through TLR-4. PLoS ONE.

[18] Manon-Jensen T (2016). Altered collagen turnover in factor VIII-deficient rats with hemophilic arthropathy identifies potential novel serological biomarkers in hemophilia. J. Thromb. Haemost..

[19] Campisi L (2016). Apoptosis in response to microbial infection induces autoreactive TH17 cells. Nat. Immunol..

[20] Brosh N, Eilat E, Zinger H, Mozes E (2000). Characterization and role in experimental systemic lupus erythematosus of T-cell lines specific to peptides based on complementarity-determining region-1 and complementarity-determining region-3 of a pathogenic anti-DNA monoclonal antibody. Immunology.

[21] Zhou Y (2022). CD4(+) T cell activation and inflammation in NASH-related fibrosis. Front. Immunol..

[22] Zhang M (2020). A STAT3 palmitoylation cycle promotes TH17 differentiation and colitis. Nature.

[23] Zhou H (2018). Protective effects the Akt activator SC79 in hepatic ischemia-reperfusion injury. Med Sci. Monit..

[24] Mishra S (2022). Sirtuin 6 inhibition protects against glucocorticoid-induced skeletal muscle atrophy by regulating IGF/PI3K/AKT signaling. Nat. Commun..

[25] Lu K (2022). Defects in a liver-bone axis contribute to hepatic osteodystrophy disease progression. Cell Metab..

[26] Yuan CH (2020). Classification of four distinct osteoarthritis subtypes with a knee joint tissue transcriptome atlas. Bone Res..

[27] Wu HJ (2010). Gut-residing segmented filamentous bacteria drive autoimmune arthritis via T helper 17 cells. Immunity.

[28] Ono-Ohmachi A (2021). Effector memory CD4(+)T cells in mesenteric lymph nodes mediate bone loss in food-allergic enteropathy model mice, creating IL-4 dominance. Mucosal Immunol..

[29] Mehrotra P, Collett JA, Gunst SJ, Basile DP (2018). Th17 cells contribute to pulmonary fibrosis and inflammation during chronic kidney disease progression after acute ischemia. Am. J. Physiol. Regul. Integr. Comp. Physiol..

[30] Snir O (2012). Multifunctional T cell reactivity with native and glycosylated type II collagen in rheumatoid arthritis. Arthritis Rheum..

[31] Inglis JJ, Šimelyte E, McCann FE, Criado G, Williams RO (2008). Protocol for the induction of arthritis in C57BL/6 mice. Nat. Protoc..

[32] Perez-Garcia S (2019). Profile of matrix-remodeling proteinases in osteoarthritis: impact of fibronectin. Cells.

[33] Wheeler K (2011). Regulatory T cells control tolerogenic versus autoimmune response to sperm in vasectomy. Proc. Natl. Acad. Sci. USA.

[34] Rao NA, Xu S, Font RL (1985). Sympathetic ophthalmia. An immunohistochemical study of epithelioid and giant cells. Ophthalmology.

[35] Stanic I (2006). Polyamine depletion inhibits apoptosis following blocking of survival pathways in human chondrocytes stimulated by tumor necrosis factor-alpha. J. Cell Physiol..

[36] Yao X (2019). Fibroblast growth factor 18 exerts anti-osteoarthritic effects through PI3K-AKT signaling and mitochondrial fusion and fission. Pharm. Res..

[37] Datta SR (1997). Akt phosphorylation of BAD couples survival signals to the cell-intrinsic death machinery. Cell.

[38] Xie J (2019). Sustained Akt signaling in articular chondrocytes causes osteoarthritis via oxidative stress-induced senescence in mice. Bone Res..

[39] Nieuwenhuizen L (2014). Hemarthrosis in hemophilic mice results in alterations in M1-M2 monocyte/macrophage polarization. Thromb. Res..

[40] Ravanbod R, Torkaman G, Esteki A (2011). Biotribological and biomechanical changes after experimental haemarthrosis in the rabbit knee. Haemophilia.

[41] Felli L (2019). Single intravenous administration of tranexamic acid in anterior cruciate ligament reconstruction to reduce postoperative hemarthrosis and increase functional outcomes in the early phase of postoperative rehabilitation: a randomized controlled trial. Arthroscopy.

[42] Wang Y (2017). Exosomes from embryonic mesenchymal stem cells alleviate osteoarthritis through balancing synthesis and degradation of cartilage extracellular matrix. Stem Cell Res. Ther..

[43] Chen Y (2022). A high-resolution route map reveals distinct stages of chondrocyte dedifferentiation for cartilage regeneration. Bone Res..

[44] Sun Q (2021). Parathyroid hormone attenuates osteoarthritis pain by remodeling subchondral bone in mice. eLife.

[45] Altman RD, Gold GE (2007). Atlas of individual radiographic features in osteoarthritis, revised. Osteoarthr. Cartil..

[46] Zhang T (2022). Systemic and single cell level responses to 1 nm size biomaterials demonstrate distinct biological effects revealed by multi-omics atlas. Bioact. Mater..

