# Supplementary material for: A pathological joint–liver axis mediated by matrikine-activated CD4+ T cells
Source: Signal Transduct Target Ther. 2024 May 8;9:109. doi: 10.1038/s41392-024-01819-y (PMC11076293; doi:10.1038/s41392-024-01819-y)
Supplement: Supplementary file 1 — Supplementary materials [file 41392_2024_1819_MOESM1_ESM.docx]

**Supplemantary Materials**

**A pathological joint-liver axis mediated by matrikine-activated CD4^+^ T cells**

Junzhi Yi, Hui Zhang, Fangyuan Bao, Zhichu Chen, Yuliang Zhong, Tianning Ye, Xuri Chen, Jingyi Qian, Mengya Tian, Min Zhu, Zhi Peng, Zongyou Pan, Jianyou Li, Zihao Hu, Weiliang Shen, Jiaqi Xu, Xianzhu Zhang, Youzhi Cai, Mengjie Wu, Hua Liu, Jing Zhou, Hongwei Ouyang

Corresponding author: hwoy@zju.edu.cn (Hongwei Ouyang), zhoujing@zju.edu.cn (Jing Zhou)

This file includes:

Supplementary figures 1-16

Supplementary tables 1-5


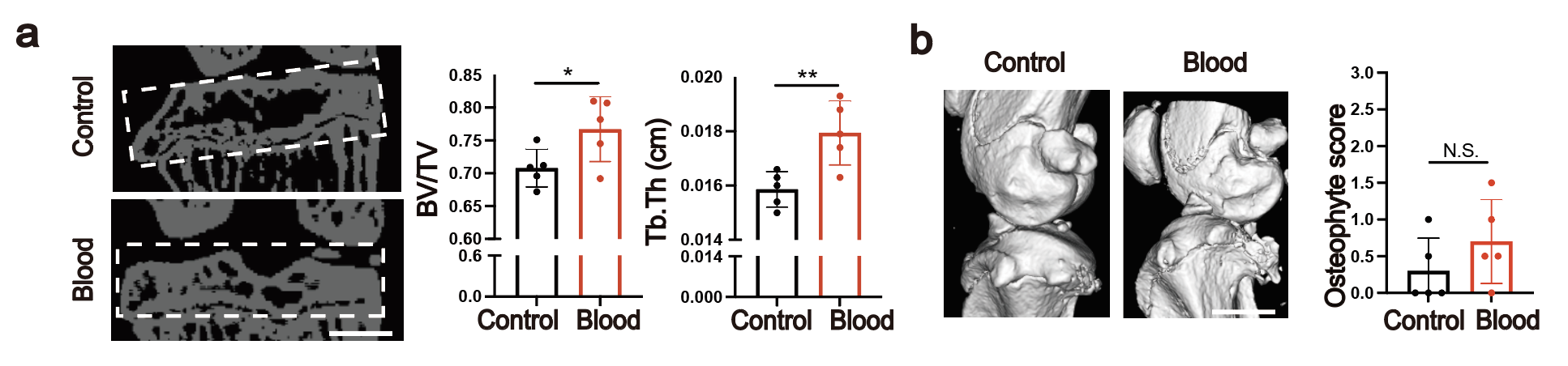


**Supplementary Fig. 1 Phenotypes of subchondral bone sclerosis and osteophyte formation after intra-articular (IA) blood treatment.**

(a) The knee joints of mice that were treated with blood or PBS were performed micro-CT. The subchondral bone sclerosis were assayed by BT/TV, Tb.Th in framed areas (n = 5) (scale bar, 600 µm). (b) After the IA blood treatment, osteophyte formation was assayed by micro-CT and evaluated by osteophyte score (n = 5) (scale bar, 1200 µm). *P < 0.05, **P < 0.01.


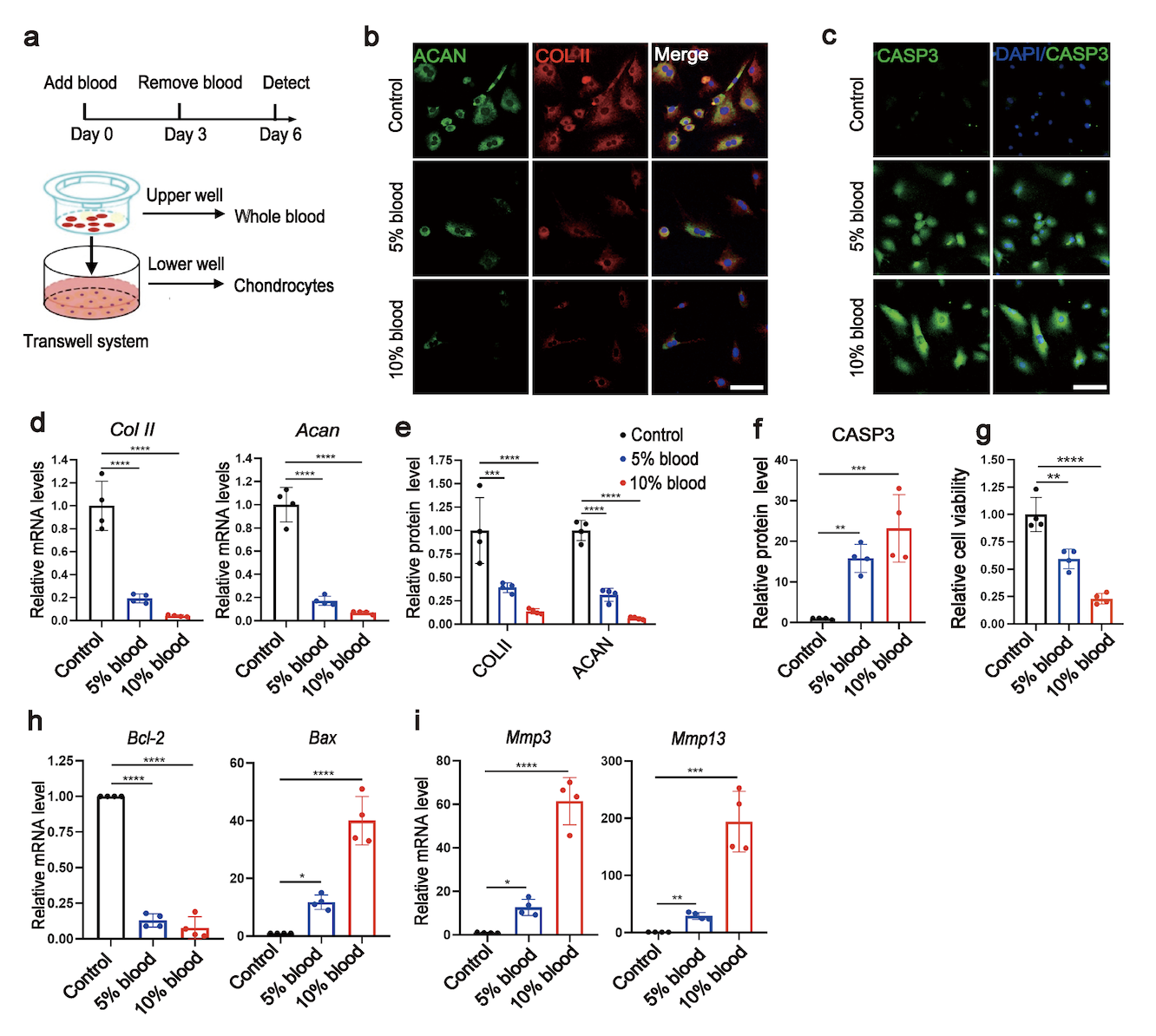


**Supplementary Fig. 2** **Blood induces chondrocyte degeneration and apoptosis.**

(a) The experimental procedure for blood treatment *in vitro*. The whole blood was added into the upper well and the chondrocytes were cultured in lower well. The blood was removed after three days. The chondrocytes were cultured for another 3 days for further detection. (b and c) Immunofluorescence analysis of ACAN (green), COL II (red), C-caspase-3 (green) in the chondrocytes treated with different concentration of blood (scale bar, 100 µm). (d) The chondrocyte mRNA expression of *Acan* and *Col II* was measured by qPCR (n = 4). (e and f) The quantitation of ACAN, COL II and CASP3 protein level from b and c, respectively (n = 4). (g) Cell viability of chondrocytes after blood treatment (n = 4). (h) The mRNA expression of apoptosis-resistant *Bcl-2* and apoptosis-promoting *Bax* in chondrocytes measured by qPCR (n = 4). (i) The mRNA expression of matrix degradation-related *Mmp3* and *Mmp13* in chondrocytes measured by qPCR (n = 4). *P < 0.05, **P < 0.01, ***P < 0.001, ****P < 0.0001.


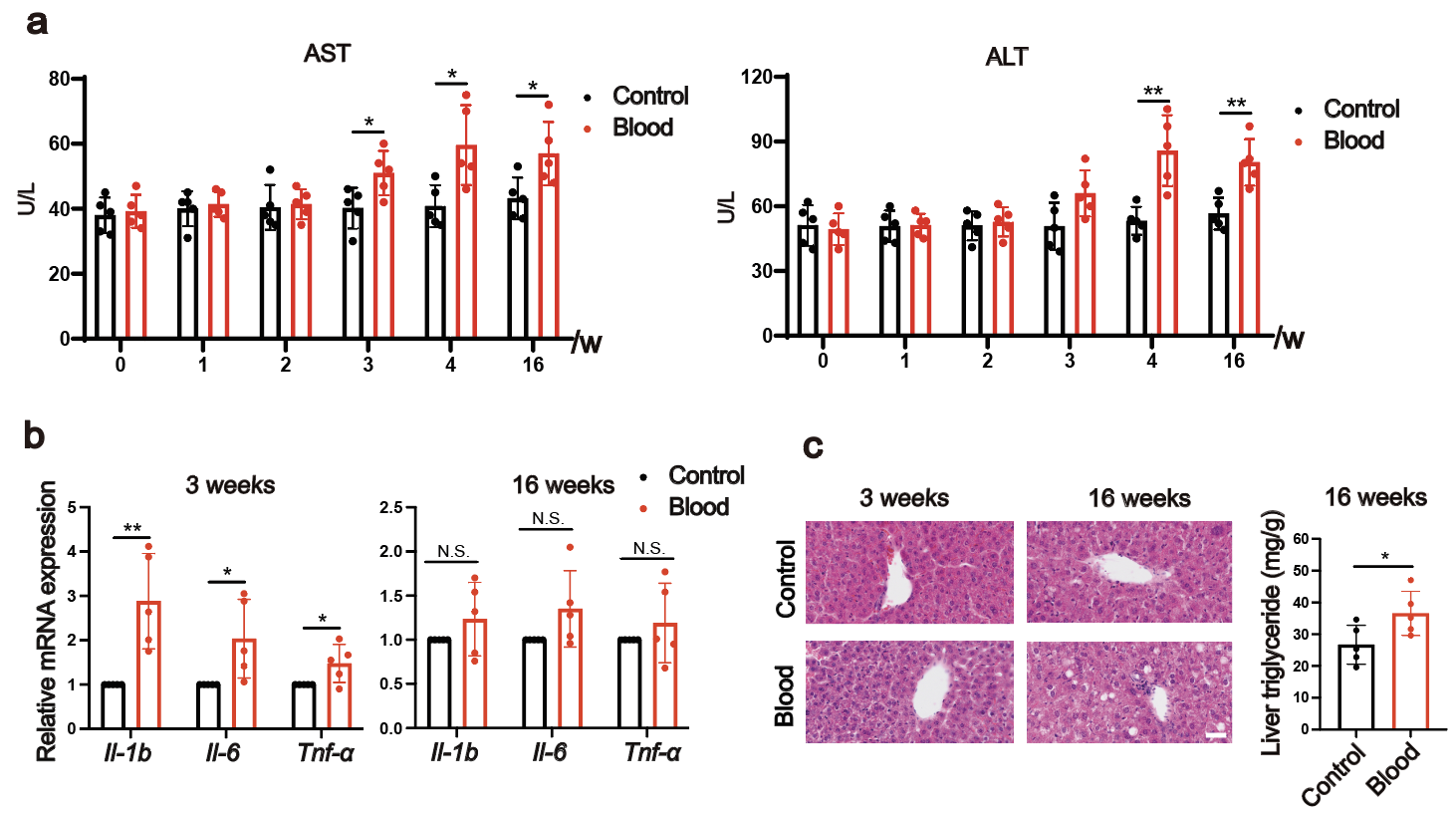


**Supplementary Fig. 3 Liver damage at different stages of pathological processes after IA blood treatment**

(a) During and after the 4-time IA blood treatment, serological AST and ALT were assayed at 0, 1, 2, 3, 4, 16 week (n = 5). (b) The liver mRNA expression of *Il-1b, Il-6 and Tnf-α* measured by qPCR at 3 and 16 weeks (n = 5). (c) HE staining of the collected liver tissues at 3 and 16 weeks (scale bar, 40 µm) and evaluation of liver triglyceride (n = 5). *P < 0.05, **P < 0.01.


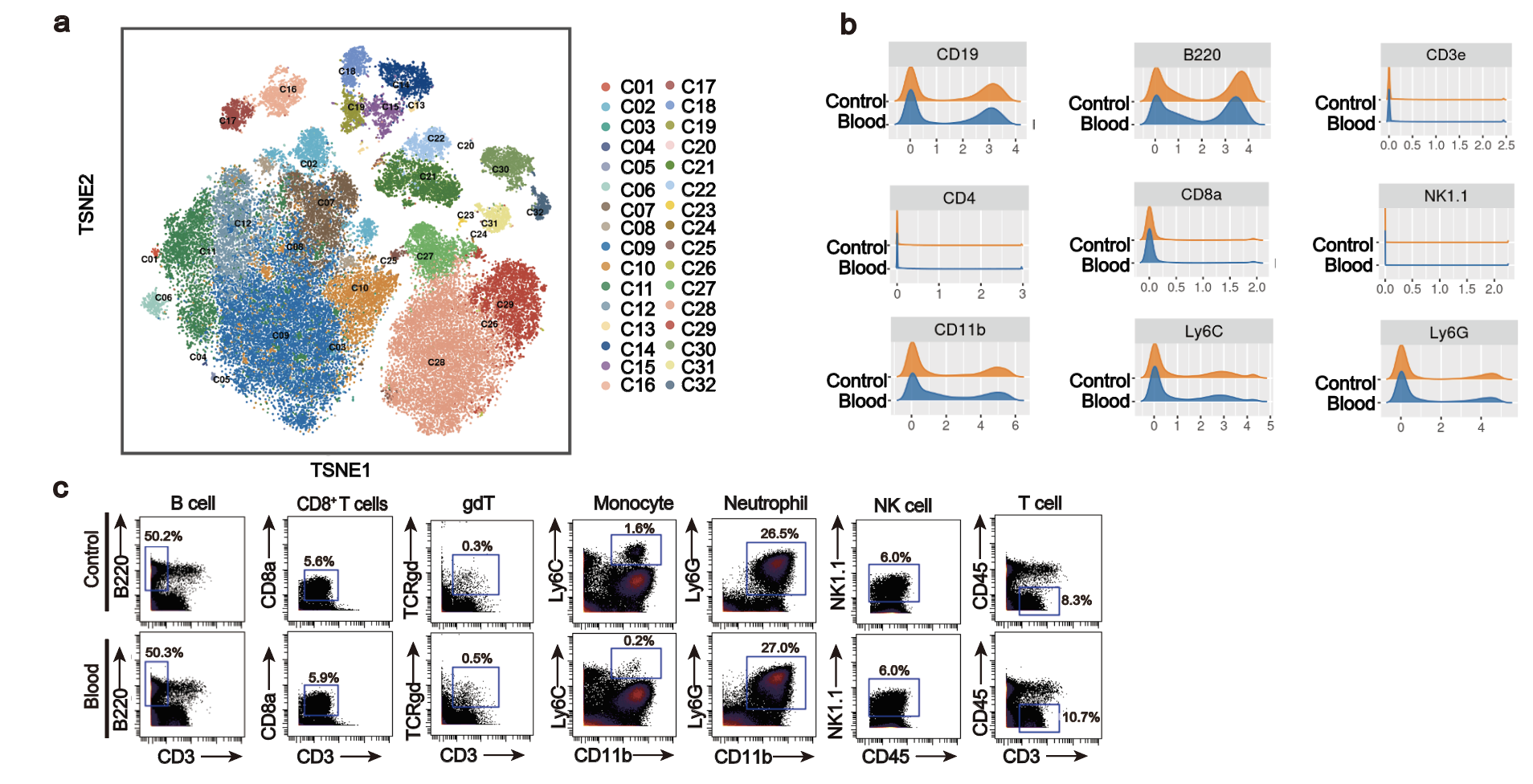


**Supplementary Fig. 4** **CyTOF analysis of peripheral CD45^+^ peripheral blood mononuclear cells (PBMCs)**

(a) t-Distributed Stochastic Neighbor Embedding (TSNE) analysis showing cell clusters from CyTOF data (b) Histogram showing the relative expression of representative markers, including B cell makers (CD19 and B220), T cell markers (CD3e, CD4 and CD8a), NK cell marker (NK1.1), myeloid cell markers (CD11b and Ly6C) and neutrophil marker (Ly6G). (c) The corresponding quantification of main cell clusters by SPADE analysis of the CD45^+^ cells. In CyTOF analysis, 136389 CD45^+^ cells from 4 donor mice in Control group and 147281 CD45^+^ cells from 4 donor mice in Blood group were measured.


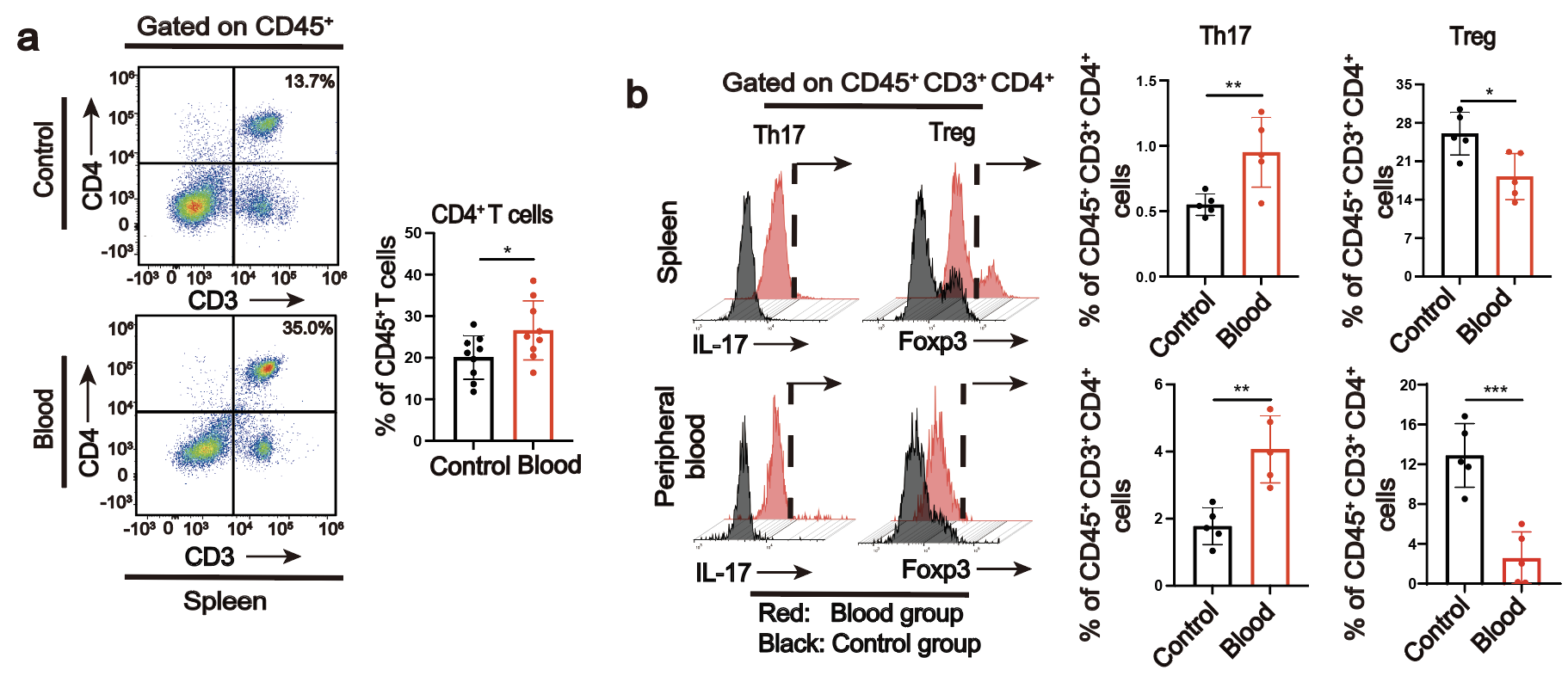


**Supplementary Fig. 5 Characterization of CD4^+^ T cells in spleen and peripheral blood after IA blood treatment**

(a) Flow cytometric measurement of the percentages of CD4^+^ T cells in spleen (n = 9). (b) Flow cytometric analysis of the percentages of Th17 and Treg cells in spleen and peripheral blood and corresponding quantitation (n = 5).


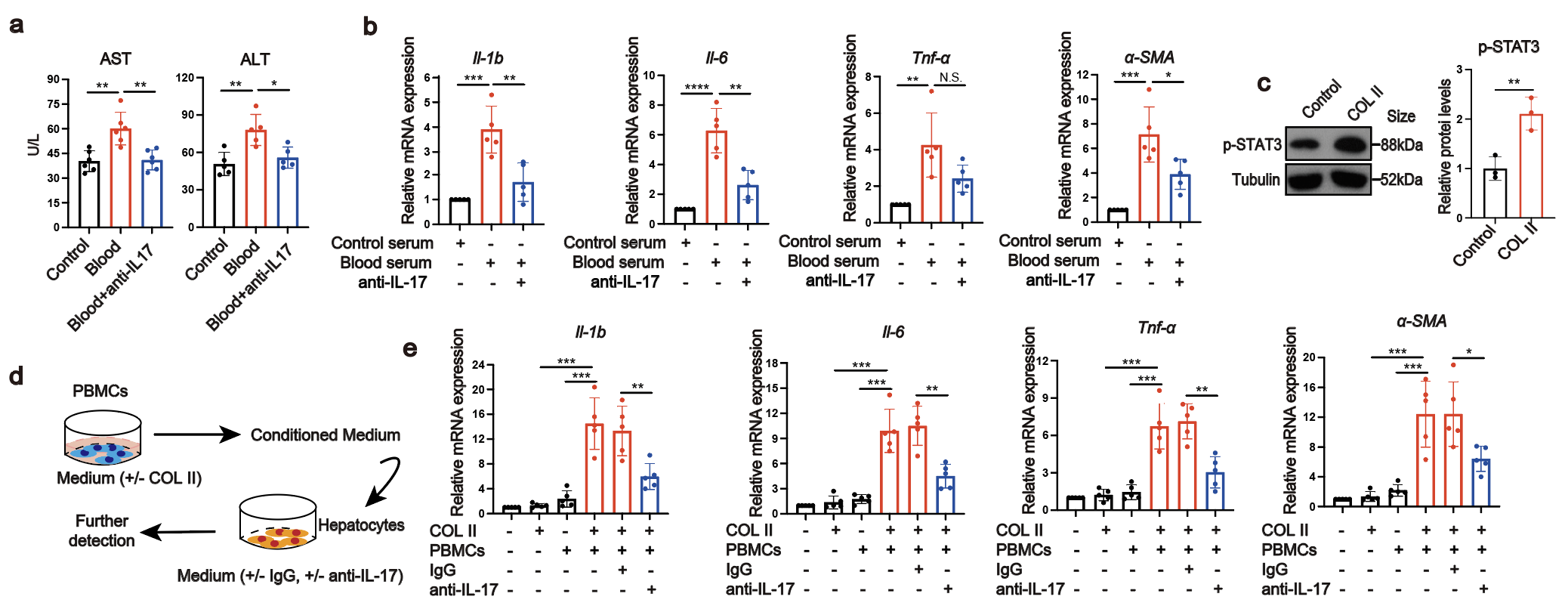


**Supplementary Fig. 6 The effects of IL-17 in hemarthrosis model and sCOL II-induced hepatocyte damage**

(a) Serological AST and ALT levels *in vivo* with or without IL-17 antibody. (b) The mRNA expression of *Il-1b, Il-6, Tnf-α* and *α-SMA* in hepatocytes measured by qPCR after treated with serum collected from Control or Blood group (with or without IL-17 antibody)(n = 5). (c) Western Blot anaylsis of p-STAT3 content in CD4^+^ T cells of “Mixed” splenocytes after sCOL II treatment and corresponding quantification (n = 3). (d) A schematic diagram of *in vitro* experiments of hepatocytes (AML12 cells) (with or without IgG, anti-IL-17) treated with conditioned medium collected from PBMCs (cultured with or without sCOL II). (e) The mRNA expression of *Il-1b, Il-6, Tnf-α* and *α-SMA* in hepatocytes measured by qPCR (n = 5). *P < 0.05, **P < 0.01, ***P < 0.001, ****P < 0.0001.


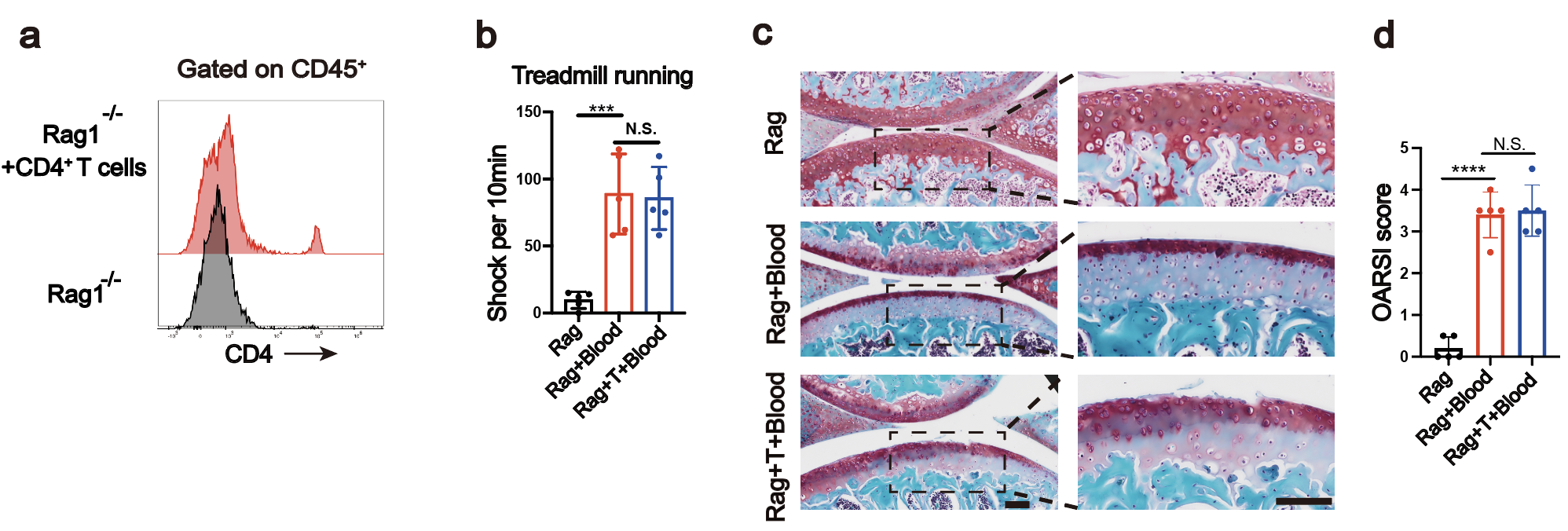


**Supplementary Fig. 7 Joint characterization of Rag1^-/-^ mice with or without adoptively transferred with CD4^+^ T cells**

(a) FACS analysis for comfirmation the succesful transfer of CD4^+^ T cells (n = 5). (b) Mice that were IA treated with blood and were performed treadmill running assay. The shock times were recorded in 10 min (n = 4). (c) SO staining of knee joints (scale bar, 100 µm). (d) Quantification of the SO staining with OARSI score (n = 5). ****P < 0.0001.


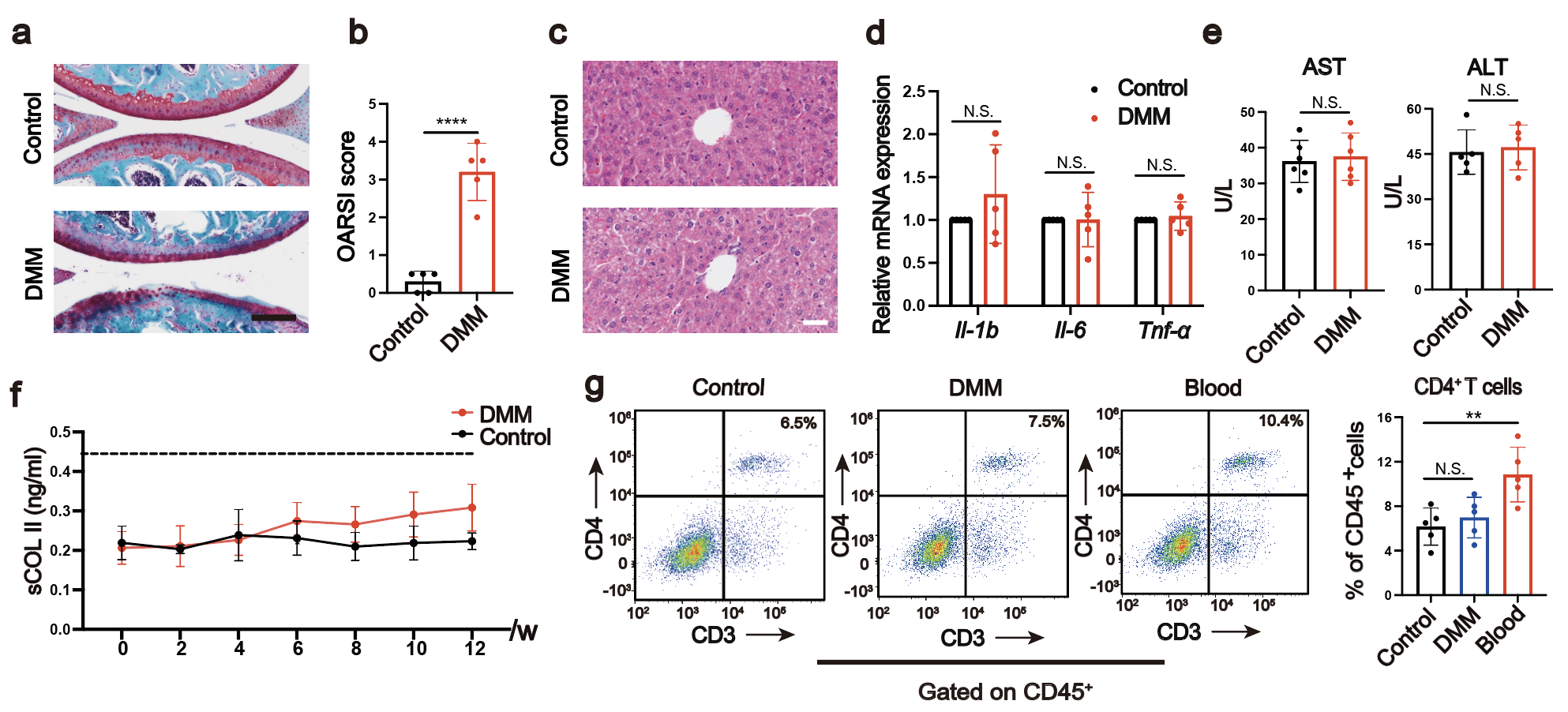


**Supplementary Fig. 8 Joint damage, liver function, serological COL II and peripheral CD4^+^ T cells of DMM model**

(a) SO staining of knee joints (scale bar, 200µm). (b) Quantification of the SO staining with OARSI score (n = 5). (c) HE staining of the collected liver (scale bar, 40 µm) (n = 5). (d) The liver mRNA expression of *Il-1b, Il-6 and Tnf-a* measured by qPCR (n = 5). (e) Serological AST and ALT levels (n = 5). (f) ELISA analysis of serological COL II level after 0, 2, 4, 6, 8, 10, 12 week after DMM was established. Dashed line indicated the level in hemarthrosis model at week 4 (1 day of the 4^th^ blood treatment) (n = 3). (g) Flow cytometric measurement of the percentages of CD4^+^ T cells in peripheral blood (n = 5). **P < 0.01, ****P < 0.0001.


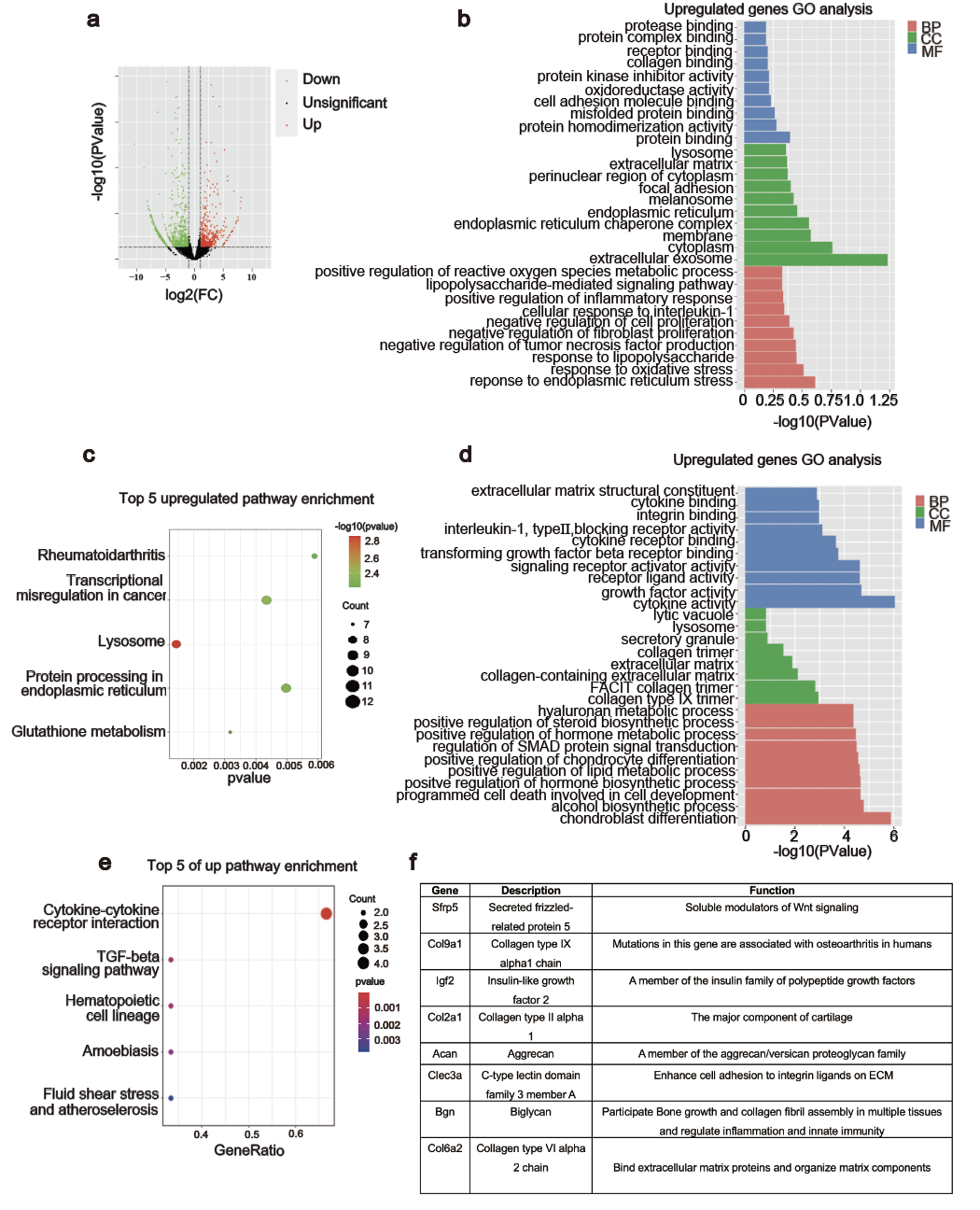


**Supplementary Fig. 9 Transcriptomic analysis of cartilage explants after blood treatment.**

(a) Volcano plot of up- and down-regulated genes in Blood group from RNA-Seq data (n = 3). (b) GO analysis of up-regulated genes in Blood group from RNA-Seq data (n = 3). (c) KEGG pathway analysis of up-regulated genes in Blood group from RNA-Seq data (n = 3). (d) GO analysis of up-regulated genes in Blood group from single-cell qPCR data. (e) KEGG pathway analysis of up-regulated genes in Blood group from single-cell qPCR data. (f) Overlapped down-regulated genes of Blood group from both RNA-Seq and single-cell qPCR data. In single-cell qPCR analysis, 36 blood-treated cells and 34 control cells were measured.


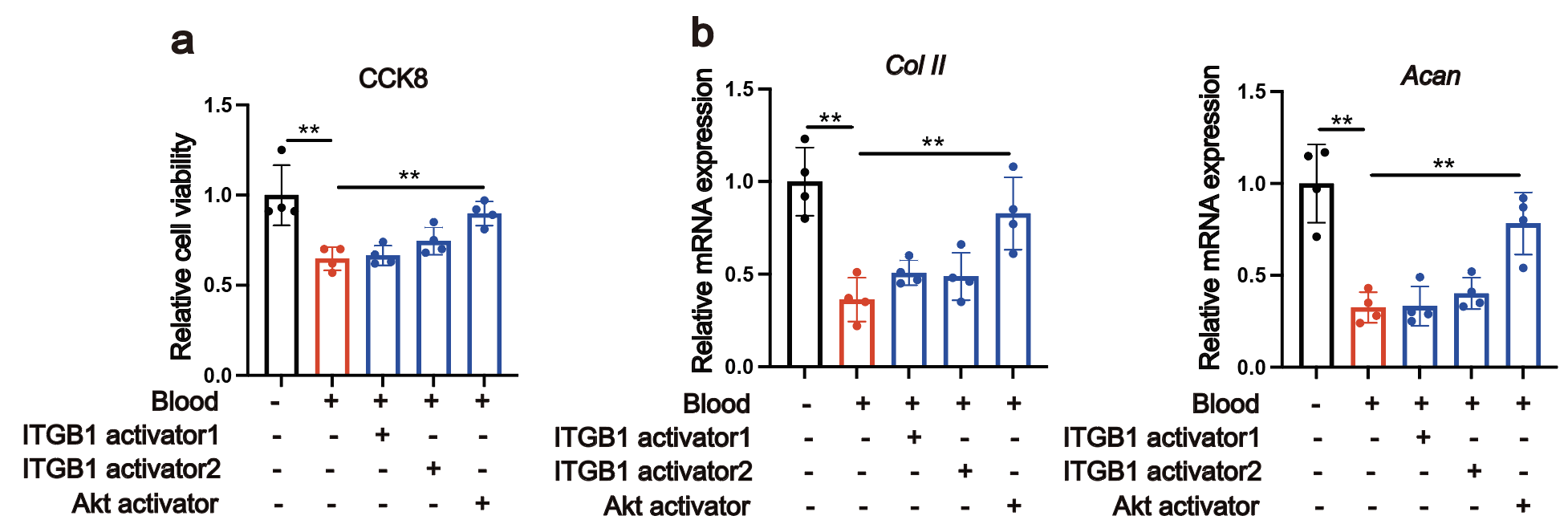


**Supplementary Fig. 10 The effects of different activators on blood-treated chondrocytes**

(a) Chondrocyte viability by CCK-8 assay (n = 4). (b)The mRNA expression of *Acan, Col II* in chondrocytes measured by qPCR (n = 4). **P < 0.01. ITGB1 activator1 (2 μM) and activator2 (10 μM) means different concentration of the activator (Pyrintegrin).


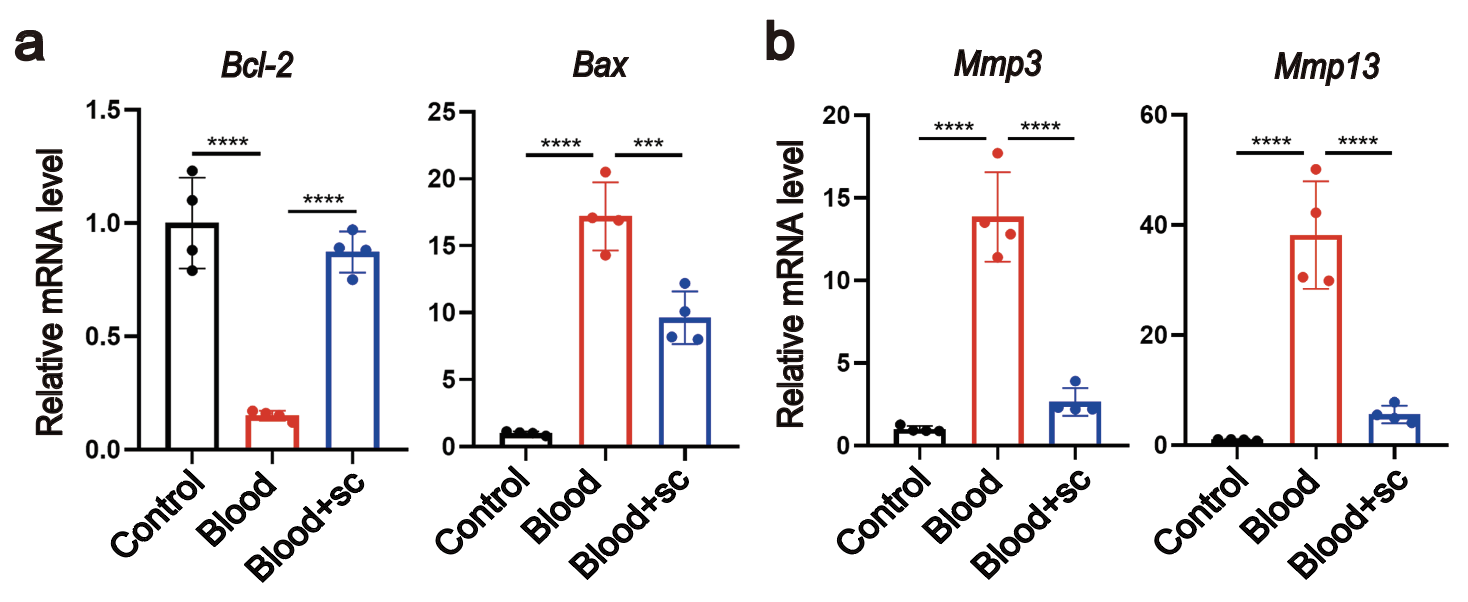


**Supplementary Fig. 11 qPCR analysis of apoptosis and matrix degradation-related genes.**

(a) The mRNA expression of apoptosis-resistant *Bcl-2* and apoptosis-promoting *Bax* in chondrocytes measured by qPCR (n = 4). (b) The mRNA expression of matrix degradation-related *Mmp3* and *Mmp13* in chondrocytes measured by qPCR (n = 4). ***P < 0.001, ****P < 0.0001.


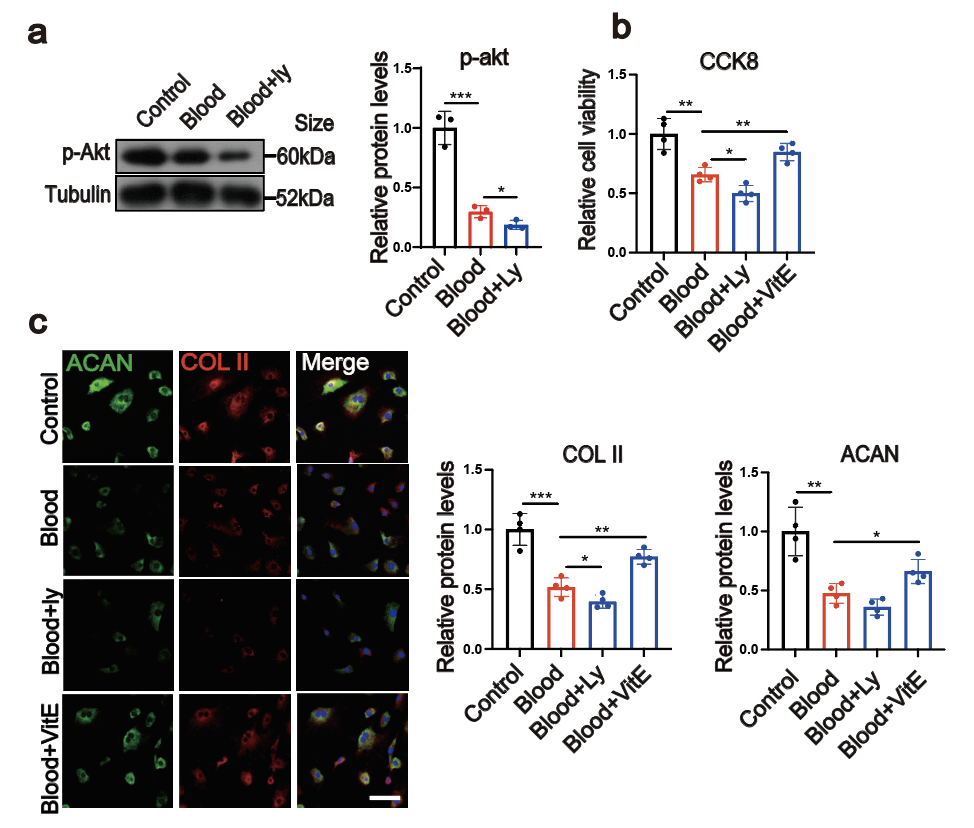


**Supplementary Fig. 12 The effects of other small molecules on Akt pathway and phenotype of chondrocytes**

(a) Western Blot analysis of p-Akt expression and quantification normalized by Tubulin on chondrocytes (n = 3). (b) Chondrocyte viability by CCK-8 assay (n = 4). (c) Immunofluorescence analysis of ACAN (green), COL II (red) in the chondrocytes (scale bar, 100 µm) and the quantitation of ACAN, COL II protein level, respectively (n = 4). *P < 0.05, **P < 0.01, ***P < 0.001


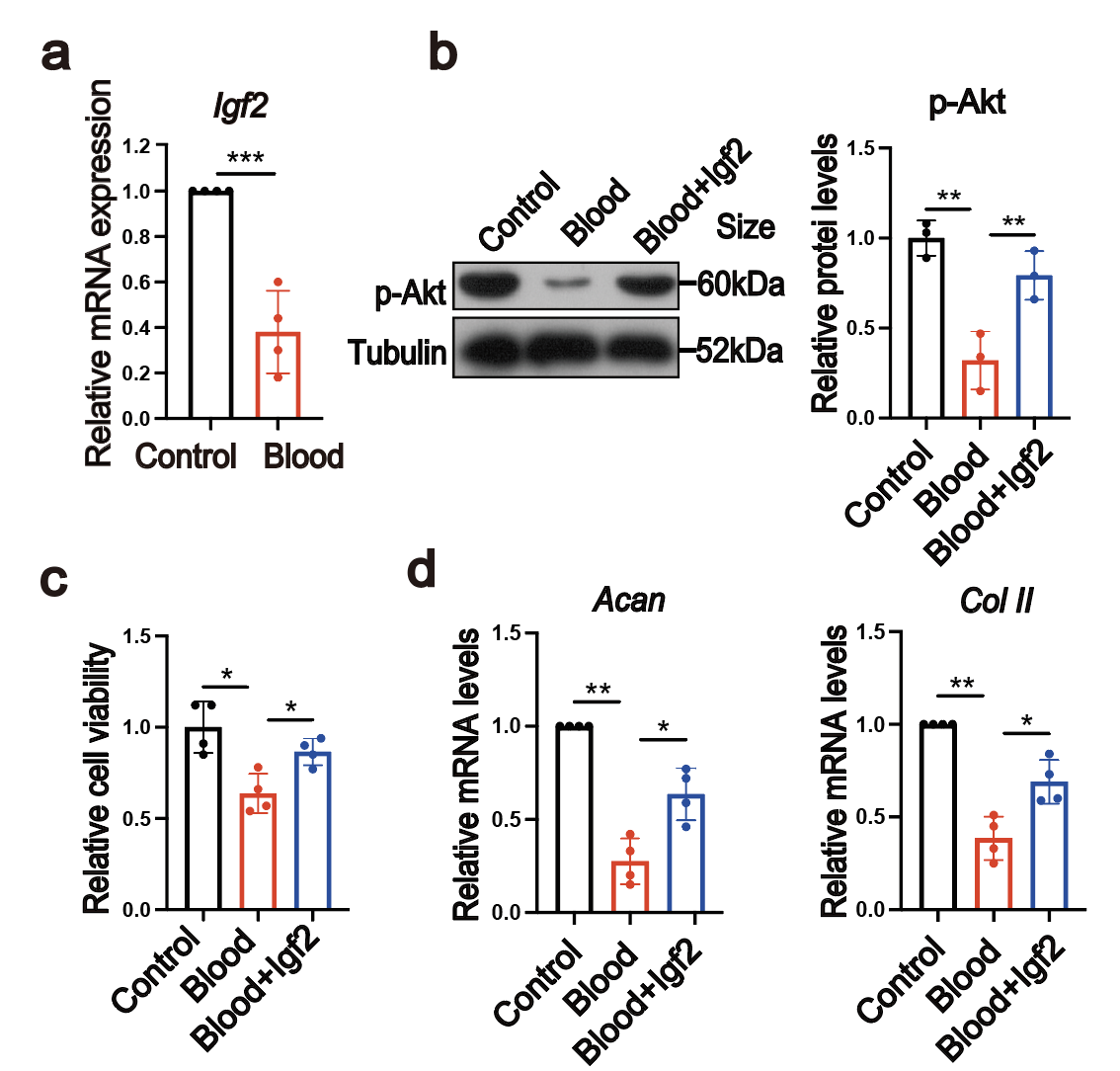


**Supplementary Fig. 13 The effects of Igf2 on the Akt pathway and phenotype of chondrocytes**

(a) qPCR analysis of *Igf2* expression in chondrocytes (n = 4). (b) Western Blot analysis of p-Akt expression and quantification normalized by Tubulin in chondrocytes (n = 3). (c) Cell viability of chondrocytes (n = 4). (d) qPCR analysis of *Acan* and *Col II* mRNA level in chondrocytes (n = 4). *P < 0.05, **P < 0.01, ***P < 0.001


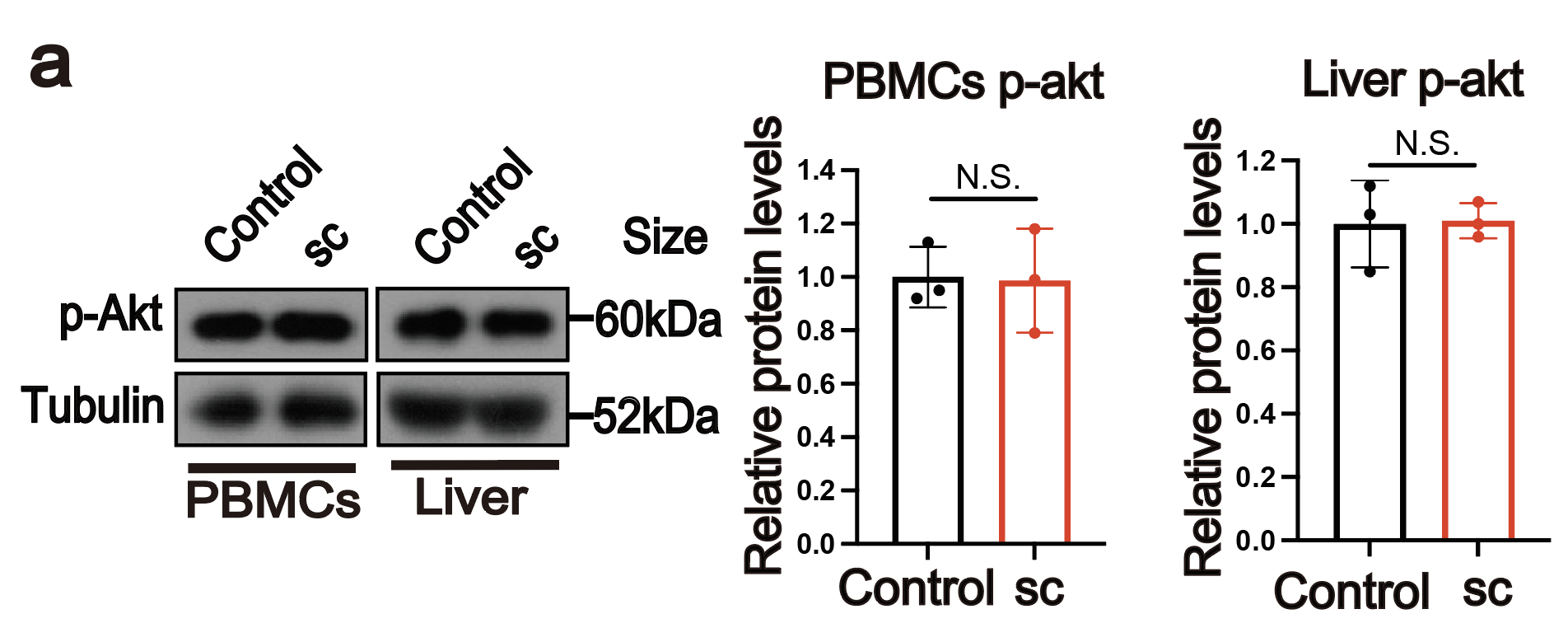


**Supplementary Fig. 14 The effects of sc79 on Akt pathway in other peripheral tissues**

(a) Western Blot analysis of p-Akt expression and quantification normalized by Tubulin on PBMCs and liver (n = 3).


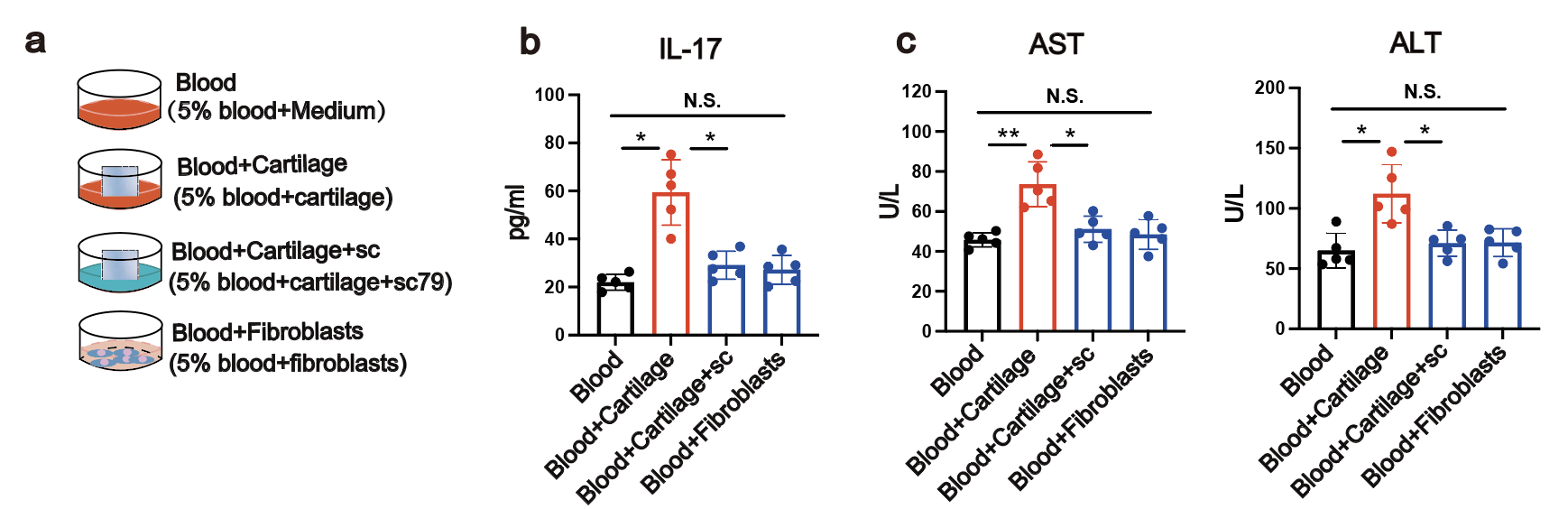


**Supplementary Fig. 15 Cartilage Akt activation protects against the peripheral damage**

(a) The group information of the co-culture system. Condition media of these co-culture systems were IV (intravenous) injected into the mice. (b) Serum IL-17 level of mice *in vivo* (n = 5). (c) Serum biochemical analysis of AST and ALT (n = 5). *P < 0.05, **P < 0.01.


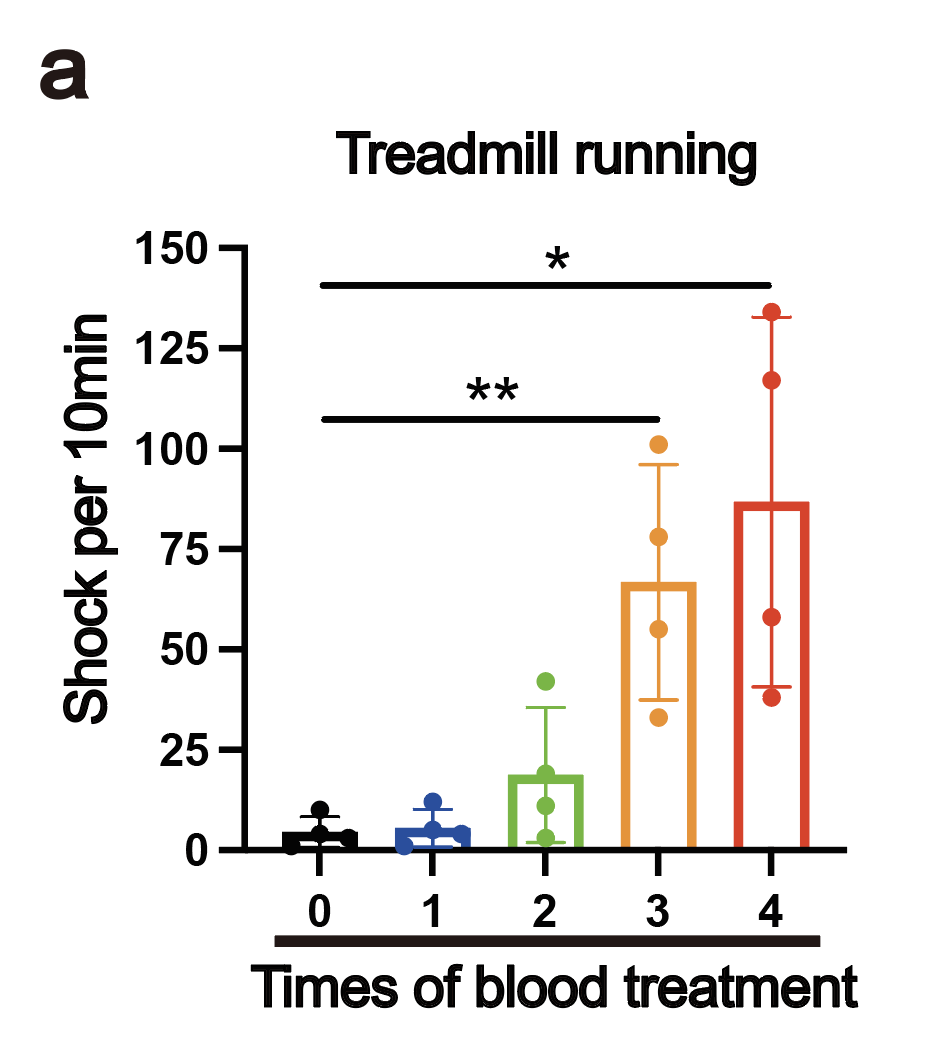


**Supplementary Fig. 16 Sports ability at different time points after blood treatment**

(a) Mice that were IA treated with 1, 2, 3 and 4 times of blood and were performed treadmill running assay. The shock times were recorded in 10 min (n = 4). *P < 0.05, **P < 0.01.

**Supplementary Table. 1 Baseline characteristics of healthy controls (HC), primary osteoarthritis (prOA) and post-traumatic joint damage (PTJD) groups**

|  | HC  (n=106) | prOA)  (n=109) | PTJD  (n=102) |
| --- | --- | --- | --- |
| Age (mean±SD),y | 62.25±6.91 | 63.77±9.67 | 61.28±9.47 |
| Median (range) | 36-80 | 18-84 | 23-83 |
| BMI (mean±SD), kg/m^2^ | 24.01±2.90 | 25.14±3.94 | 24.76±3.51 |
| Sex, n(%) |  |  |  |
| Men | 53(50) | 55(50.46) | 51(50) |
| Women | 53(50) | 54(49.54) | 51(50) |
| Time interval (months) | / | 74.54±72.10 | 57.76±116.41 |
| Median (range) | / | 0.1-360 | 0-600 |

**Supplementary Table 2 qPCR primers**

| Genes | Species | Direction | Sequence (5’-3’) |
| --- | --- | --- | --- |
| *Bcl-2* | Mouse | F | GCTACCGTCGTGACTTCGC |
|  |  | R | CCCCACCGAACTCAAAGAAGG |
| *Bax* | Mouse | F | AATTCGCCGGAGACACTCG |
|  |  | R | CCAGCCCATGATGGTTCTGAT |
| *Col 2a1* | Mouse | F | TGGTGCTCGGGGTAACGAT |
|  |  | R | GGCTCCAGGAATACCATCAGT |
| *Acan* | Mouse | F | GTGGAGCCGTGTTTCCAAG |
|  |  | R | AGATGCTGTTGACTCGAACCT |
| *Mmp3* | Mouse | F | GGCCTGGAACAGTCTTGGC |
|  |  | R | TGTCCATCGTTCATCATCGTCA |
| *Mmp13* | Mouse | F | TGTTTGCAGAGCACTACTTGAA |
|  |  | R | CAGTCACCTCTAAGCCAAAGAAA |
| *Il-1b* | Mouse | F | GAAATGCCACCTTTTGACAGTG |
|  |  | R | TGGATGCTCTCATCAGGACAG |
| *Il-6* | Mouse | F | CTGCAAGAGACTTCCATCCAG |
|  |  | R | AGTGGTATAGACAGGTCTGTTGG |
| *Tnf-α* | Mouse | F | CAGGCGGTGCCTATGTCTC |
|  |  | R | CGATCACCCCGAAGTTCAGTAG |
| *Gapdh* | Mouse | F | TGACCTCAACTACATGGTCTACA |
|  |  | R | CTTCCCATTCTCGGCCTTG |
| *α-SMA* | Mouse | F | CCCAGACATCAGGGAGTAATGG |
|  |  | R | TCTATCGGATACTTCAGCGTCA |

**Supplementary Table 3 Antibodies for WB, IF, IHC and Flow Cytometry (Flow Ct).**

| Protein | Source | Application |
| --- | --- | --- |
| ACAN | Santa Cruz, sc-33695 | 1:200 (IF) |
| COL II | Sigma, SAB4500366 | 1:200 (IF) |
| Cleaved caspase-3 | CST, 9661 | 1:200 (IF) |
| α-SMA | Abcam, ab124964 | 1:500 (IHC) |
| p-Akt | CST, 4060 | 1:3000 (WB) |
| Tubulin | CST, 2125 | 1:8000 (WB) |
| p-STAT3 | Abcam, ab76315 | 1:3000 (WB) |
| CD3-FITC | Invitrogen, 11-0031-82 | 1:50 (Flow Cyt) |
| CD4-PE | Invitrogen, 12-0041-82 | 1:50 (Flow Cyt) |
| CD45-APC | eBioscience, 17-0454-811 | 1:50 (Flow Cyt) |
| CD3-APC-Cy7 | Biolegend, 100221 | 1:50 (Flow Cyt) |
| IL17-FITC | eBioscience, 11-7177-80 | 1:50 (Flow Cyt) |
| Foxp3-FTIC | Biolegend, 126405 | 1:50 (Flow Cyt) |
| Anti-rat HRP | Abcam, ab6734 | 1:1000 (IHC) |
| Anti-rabbit HRP | Jackson, 111-035-003 | 1:1000 (WB) |
| Anti-rabbit 546 | Invitrogen, A11035 | 1:1000 (IF) |
| Anti-mouse 488 | Invitrogen, A21202 | 1:1000 (IF) |

**Supplementary Table 4 Cytof antibodies**

| List | Label | Marker | clone | Category |
| --- | --- | --- | --- | --- |
| 1 | 89Y | CD45 | 30-F11 | surface marker |
| 2 | 115In | CD3e | 145-2C11 | surface marker |
| 3 | 141Pr | CD117(c-kit) | 2B8 | surface marker |
| 4 | 142Nd | MHC II(I-A/I-E) | M5/114.15.2 | surface marker |
| 5 | 143Nd | CD45R(B220) | RA3-6B2 | surface marker |
| 6 | 144Nd | Gr-1(Ly-6G/Ly-6C) | RB6-8C5 | surface marker |
| 7 | 145Nd | CD80 | 16-10A1 | surface marker |
| 8 | 146Nd | CD38 | 90 | surface marker |
| 9 | 147Sm | CD161(NK-1.1) | PK136 | surface marker |
| 10 | 148Nd | Ly-6C | HK1.4 | surface marker |
| 11 | 149Sm | CD19 | 6D5 | surface marker |
| 12 | 150Nd | CD127(IL-7Rα) | A7R34 | surface marker |
| 13 | 151Eu | CD62L | MEL-14 | surface marker |
| 14 | 152Sm | CD11c | N418 | surface marker |
| 15 | 153Eu | CD44 | IM7 | surface marker |
| 16 | 154Sm | T-bet | 4B10 | Intracellular marker |
| 17 | 155Gd | CD317(BST-2,Tetherin) | 44E9R | surface marker |
| 18 | 156Gd | FOXP3 | FJK-16s | Intracellular marker |
| 19 | 157Gd | FcεRIα | MAR-1 | surface marker |
| 20 | 158Gd | TCR γ/δ | UC7-13D5 | surface marker |
| 21 | 159Tb | CD86 | GL-1 | surface marker |
| 22 | 160Gd | TCR β chain | H57-597 | surface marker |
| 23 | 161Dy | CD64(FcγRI) | X54-5/7.1 | surface marker |
| 24 | 162Dy | CD69 | H1.2F3 | surface marker |
| 25 | 163Dy | CD25 | 3C7 | surface marker |
| 26 | 164Dy | RORγ(RORC) | 600214 | Intracellular marker |
| 27 | 165Ho | Ly-6G | 1A8 | surface marker |
| 28 | 166Er | CD27 | LG.3A10 | surface marker |
| 29 | 167Er | CD206(MMR) | C068C2 | Intracellular marker |
| 30 | 168Er | IL-17A | TC11-18H10.1 | Intracellular marker |
| 31 | 169Tm | Ki-67 | SolA15 | Intracellular marker |
| 32 | 170Er | IgD | 11-26c.2a | surface marker |
| 33 | 171Yb | CX3CR1 | SA011F11 | surface marker |
| 34 | 172Yb | CD279(PD-1) | 29F.1A12 | surface marker |
| 35 | 173Yb | CD172a(SIRPα） | P84 | surface marker |
| 36 | 174Yb | CD192(CCR2) | 475301 | surface marker |
| 37 | 175Lu | Siglec-F | E50-2440 | surface marker |
| 38 | 176Yb | MERTK(Mer) | 2B10C42 | surface marker |
| 39 | 197Au | CD4 | RM4-5 | surface marker |
| 40 | 198Pt | CD8a | 53-6.7 | surface marker |
| 41 | 209Bi | CD11b | M1/70 | surface marker |

**Supplementary Table 5 Single-cell qPCR primers**

| **Gene** | **Forward** | **Reverse** |
| --- | --- | --- |
| *Gapdh* | ATACGGCTACAGCAACAGGG | TGTGAGGGAGATGCTCAGTG |
| *Epyc* | GACTGGATGGAAACCCTATCAA | ATAAAACTCCCAATGGGCAAAC |
| *Col10a1* | GAATTTCTGTGCCAGGAAAACC | TTTTCACCTCTTCTTCCCACTC |
| *Nupr1* | GGATGAAGATGGAATCCTGGAT | TTTCCTTTCAGAGTTCTGGAAC |
| *Rpl32p* | CTCACAGAGCTAGCACATGATA | CTCCTACATCGGTTAACTTCGA |
| *Manf* | ACCAAGATCATCAATGAGGTGT | ATCTGGCTGTCTTTCTTCTTCA |
| *Itm2a* | TTCTGAGGATCCTGTCAATTCC | TCAAAGTCGTGAATAATTGCCG |
| *Sparc* | TATGCAGCAATGACAACAAGAC | TATTTGCATGGTCCGATGTAGT |
| *Bgn* | TCTATCTGCACTCCAACAACAT | GATGCCATTATAGTAGGCCCTC |
| *Clec3a* | AAATCAATGCCCTTCGAGACTA | GACCATGTCATTTATGCCTAGC |
| *Comp* | ACTGCCTGCGTTCTAGTGC | CGCCGCATTAGTCTCCTGAA |
| *Igf2* | GTTGGTGCTTCTCATCTCTTTG | AAACTGAAGCGTGTCAACAAG |
| *Fmod* | CACAACAGTCTCACTAACAACG | TCTTCTGCAGCTGATTGTAAGA |
| *Acan* | AGGTCTGTGCCATCTGTGAG | CCGAGAAATGACACCTGCTA |
| *Sox9* | GAGGCCACGGAACAGACTCA | CAGCGCCTTGAAGATAGCATT |
| *Matn3* | AGATAGAGTTCCAGCTCAACAC | CCCATCTGTCACGATAATAGCT |
| *Col11a2* | GACTGTAAGAAGCGAGTTACCC | AAAGATCACCACCCCGTG |
| *Ptpn14* | ACAACAGCCTGGACCTTAATTA | CTCATGACCGTCTCGTAATCTG |
| *Igfbp7* | CAAGGTCCTTCCATAGTGACG | TTACCTTGTTCCAGATGAGGAC |
| *Fn1* | CTATAGGATTGGAGACACGTGG | CTGAAGCACTTTGTAGAGCATG |
| *Sox5* | AACCACATTGCGGGACAAG | CGGCTCACCACAGTCTGTTG |
| *Rpl10aps1* | AATTCCAAATACCGAAACCTGC | AGGAAAGGAACGCTCTTTAAGA |
| *Klf4* | ACCTCCTGGACCTAGACTTTAT | GAAGACGAGGATGAAGCTGAC |
| *Col6a2* | AGTTCGTACCGCAGTTTATCAG | CCTTGTAGGCTCTTAGTGAAGG |
| *Rpl34-ps1* | CTTTCCTACAACACAGCCTCTA | ACAGTCTCATAAGGACTTTGGG |
| *Myl12a* | AAAAACCCAACTGACGAATACC | ATGGTGAGGAACATGGTGAAAT |
| *Casp8* | ACCAAATGAAGAACAAACCTCG | CTTCATTTTTCGGAGTTGGGTT |
| *Rpl37rt* | TGAAGCTACCAGAAGTCATGTT | CTTTCACTTCATAAACGTGGCA |
| *Col9a1* | ATTCAAGGTTCCCGAGGAATTC | GTATTCCATCTCGGCCATCTAC |
| *Spp1* | AAACACACAGACTTGAGCATTC | TTAGGGTCTAGGACTAGCTTGT |
| *Atf4* | AGTTTAGAGCTAGGCAGTGAAG | CATACAGATGCCACTGTCATTG |
| *Rpl14-ps1* | GAATGACCCCAAAGTTTGACTC | GCTGGTCGGGATAGGTATTTTA |
| *Sox6* | CTGGCTGGGAACGACATGAT | TCGTCATAGGCTTCCATTTCATC |
| *Peg3* | GTTATGAAGGGAGCGCATTTAG | CTCATAGCCTGTCTCATCTCAG |
| *Col2a1* | CCACACCAAATTCCTGTTCA | ACTGGTAAGTGGGGCAAGAC |
| *Adamts4* | GCCCATGGACTGGGTTCCGCGC | CGGGGCTCCAATACGTAGTAGT |
| *Runx2* | CCTTCAAGGTTGTAGCCCTC | GGAGTAGTTCTCATCATTCCCG |
| *Sfrp5* | GAGATCAAGATAGACAACGGGGA | TTGCGCTTTAAGGGGCCTG |
| *Il1r-1* | GATACAACTGATCTGCAACGTC | ACACAGATAAACGGATAGCGAT |
| *Fth1* | TAAAGAAACCAGACCGTGATGA | ATTCACACTCTTTTCCAAGTGC |
| *Sqstm1* | GAACACAGCAAGCTCATCTTTC | AAAGTGTCCATGTTTCAGCTTC |
| *B2m* | GAACTGCTACGTAACACAGTTC | GTATGTATCAGTCTCAGTGGGG |
| *Eif4g2* | GAACGACATGATGCAATCTTCA | CAAGGCATAGCTTGTCAAACTT |
| *S100b* | CACAGTTGGCAACCTTTATGAA | TCATACACTCCTCAAAGCTCAG |
| *Lcn2* | CGCTACTGGATCAGAACATTTG | CTTGCACATTGTAGCTCTGTAC |
| *S100a6* | CCATCTTCCACAAGTACTCTGG | TACTTCCTGATCCTTGTTACGG |
| *IL6r* | GCGTTTCACAGCTTAAAAATGG | TGAACTCCTTTGACCATACAGG |
| *Aldoa* | TTGACTTTCTCCTATGGTCGAG | CCACTTGGGGTATACTTTCCTT |
| *Il1r-2* | TCTGGTACCTACATTTGCACAT | CTGTATCTTTCCATCAGCGTTG |
| *Slc2a1* | GCAGTTCGGCTATAACACTGG | GCGGTGGTTCCATGTTTGATTG |
| *Eef1a1* | GGCTTCAATGTAAAGAACGTGT | CATCTTCCAGCTTCTTACCAGA |
| *Ssr3* | CTTACTTAGTAGCCTTCGCGTA | GTTTACTGTGGGGTTGAAGTTC |
| *Calr* | GTCGAATCCAAACATAAGTCCG | CGATATTCTGCTCATGCTTCAC |
| *Pdia6* | GCGGTCTGTATTCTTCTAGTGA | ACTGATGTTTGTCTGCATTGAC |
| *Rpl4* | GGAACTTCAGGAGAATCAGCTA | TGGGCAACATGTAGTAGTAACT |
| *Rps15* | TCCTCTGCGTTCATCAAAGAAT | GGCCATTTCCAAAGACAAGATT |
| *Sod1* | TGTCCATTGAAGATCGTGTGAT | TCATCTTGTTTCTCATGGACCA |
| *Nedd4* | GGAAGGACCTACTACGTAAACC | TCATCATTGTCTTCATCCGTGA |
| *Sptlc1* | GATACTGCTTTTCTGCTTCGTT | CACTTTTAAACCCGAAACACCT |
| *Cat* | CACCTTCAAGTTGGTTAATGCA | CATGACCTGGATGTAAAACGTC |
| *Papss2* | CTTGCAAACTCTACACTTCGAC | CCTTCGTACATCAAGGCAAATT |
| *Scd2* | TGTCTCTTCGCGTATTTGTACT | GTGTTTCTGAGAACTTGTGGTG |
| *Calu* | CATGAAAGACATAGTCGTGCAG | GGTCTGACTCATAGACCAGATG |
| *Sox11* | GATGAAGACGACGACGAAGAT | TTCTTGAAGCTGTAGTAGAGCC |
| *Col27a1* | CCTGAAGGCATGAAGGGTAAG | TCTCCGACAAGACCAATGAATC |
| *Cilp-2* | GACAAGTACGAGTACGACGTG | TTGTGTGAACGCACCATGTA |
| *Ucma* | GAATCTGATGCCTCCAATTTCC | TCCTTTGCTCCTCGTAATACTC |
| *Il6* | CTGCAAGAGACTTCCATCCAG | AGTGGTATAGACAGGTCTGTTGG |
| *Fkbp9* | CGGACTATCCAAGTATCGGATT | CACGTACGTGTCATAGGTTTTC |
| *Hspg2* | GTTGTCTTCCATGACGTCAAC | TAACTGCTGCTCTCGGAATCTT |
| *Plod2* | CCATATTTTCTGAACGAGCCTG | CAGTCGGGACATTTTCATAACC |
| *Col1a2* | GCCTAGCAACATGCCAATATTT | GAATACTGAGCAGCAAAGTTCC |
| *Col9a2* | ATCAAAGGAGATAAGGGTTCCC | GACTCCTTGCTGTCCCTTAAG |
| *Col1a1* | TGAACGTGGTGTACAAGGTC | CCATCTTTACCAGGAGAACCAT |
| *Gnas* | TGAATCTGAGTCTGATCACGAG | ATTTCGGTCTCGGATTCGATAT |
| *Mgp* | CTACGAATCTCACGAAAGCATG | TGGACTCTCTTTTGGGCTTTAG |
| *Mmp3* | TGTCACTGGTACCAACCTATTC | TCTCAGGTTCCAGAGAGTTAGA |
| *Rcn3* | AAGAGTATATCGCGGACCTGTA | CATGTTCCAGTTGCTCAAGATC |
| *Mmp13* | CTTTTCCTCCTGGACCAAACT | TCATGGGCAGCAACAATAAA |
| *Col11a1* | GATCGCCCGTTTTTCTATTTGA | GTCACAGTTTTCTTCTCCACAC |
| *prg4* | CACACCTTCAGGATCCATTACT | CCTCTCCACATTGTACTCACTT |
| *Il-1b* | GAAATGCCACCTTTTGACAGTG | TGGATGCTCTCATCAGGACAG |
| *Nrep* | CGTTTGCATACAAGGAAATGGA | CATCTTCTTTCGGTTCACTTCC |
| *Creb3L2* | CTGACAGAAGAGGAGAAGAGGA | GCCTTCTCCTCAGATTTTGTCA |
| *Fgf2* | AGTTGTGTCTATCAAGGGAGTG | CATTGGAAGAAACAGTATGGCC |
| *Gdf5* | CACCATCACCAGCTTTATTGAC | CTGATGTCAAACACGTACCTCT |
| *Col9a3* | CAAAGATGGTGAGAAGGGTAGT | CTGGAAGTCCTCTTAATCCTCG |
| *Adamts5* | CAGTGTGAAGCCAAAAATGGCTATC | TGCTGTACGGCCTGCATTCAGTCCC |
| *Lect1* | AGAGAGAGAGAAGAGAAGTCGT | TCTAGGGTCAAATGTCATGCTT |
| *Bmp6* | CTTCATGGTGGCCTTCTTCAAG | CTGTTTTTAACTCACTGCCGTT |
| *Hapln1* | GGCTAGAAGATGATACTGCTGT | AAGTTAAGATTGTAGCGTCCCA |
| *Fkbp10* | CACCTACAATACCTATGTCGGG | CGATTTTGGATGTCTCATTGCA |
| *Casp3* | GAAACTCTTCATCATTCAGGCC | GCGAGTGAGAATGTGCATAAAT |
| *Eef2* | CAATGTGAAGAATGTGTCCGTT | TCAATCTTCTCCTTTAGCTCGG |
| *C1qtnf3* | GAGGACGTAGAGGAAGTGTATG | TGGTTGCTGGATGTATCTGATT |
| *Smoc2* | GGTCTTCCATTTGGTGTAACAC | ATTCCAGAATGCAATGATGACG |
